# PHGDH inhibition overcomes dabrafenib resistance through metabolic rewiring in BRAF V600E anaplastic thyroid carcinoma

**DOI:** 10.1038/s41420-026-03293-7

**Published:** 2026-08-15

**Authors:** Sung Young Kim, Mi-Hyeon You

**Affiliations:** 1 Department of Biochemistry, Konkuk University School of Medicine, Seoul, Republic of Korea; 2 Department of Anatomy, College of Medicine, KonKuk University, Chungju-si, Republic of Korea

**Keywords:** Cancer metabolism, Cancer therapy

## Abstract

Dabrafenib, a BRAF kinase inhibitor, is used clinically to treat anaplastic thyroid carcinoma (ATC) harboring the BRAF V600E mutation. However, its clinical efficacy is limited due to the rapid emergence of resistance to treatment. In this study, we established a dabrafenib-resistant ATC cell line (8505C-R) and compared its molecular and phenotypic characteristics with those of the parental cells. We evaluated cell proliferation, colony formation, stem cell marker expression, and MAPK pathway activity and identified elevated levels of EGFR and PHGDH in 8505C-R cells as hallmarks of resistance. RNA sequencing revealed that PHGDH inhibition using the selective inhibitor NCT-503 profoundly reprogrammed gene expression networks related to tumor plasticity, stem-like phenotypes, and hyperactivation of the EGFR–MAPK signaling axis. Notably, LRIG1, a negative regulator of EGFR, was differentially expressed upon PHGDH inhibition, suggesting a feedback loop connecting serine metabolism with receptor tyrosine kinase signaling. Functional assays demonstrated that PHGDH inhibition, either alone or in combination with dabrafenib, markedly suppressed colony formation, expression of stemness-associated markers, and MAPK pathway activity. These results suggest that PHGDH-dependent serine metabolism is associated with resistance-related signaling changes involving the EGFR–MAPK axis in BRAF-mutant ATCs.

## Introduction

The B-Raf proto-oncogene (BRAF) V600E mutation is frequently observed in thyroid cancer, especially in anaplastic thyroid carcinoma (ATC), and Dabrafenib, a BRAF inhibitor, is approved and used for the treatment of ATC [1]. However, rapid development of resistance through compensatory activation of the epidermal growth factor receptor (EGFR) pathway has been reported when dabrafenib is used as monotherapy, which limits the clinical efficacy of the treatment [2]. EGFR is a tyrosine kinase receptor that induces cell proliferation, invasion, and angiogenesis through the protein kinase R (PKR)-like endoplasmic reticulum kinase (ERK) and protein kinase B (AKT) pathways, and combined inhibition of BRAF and EGFR has been proposed as an effective method for overcoming resistance in various cancers [3]. Although the synergy in this combination therapy has been demonstrated in colon cancer and melanoma, research on this topic in thyroid cancer including ATC remains limited [4].

In this study, we observed a significant increase in the expression of phosphoglycerate dehydrogenase (PHGDH), a key enzyme in serine/glycine metabolism, along with sustained activation of phosphorylated EGFR in dabrafenib-resistant ATC cells (8505C-R) [5]. Our findings align with recent studies demonstrating that PHGDH overexpression contributes to resistance mechanisms, suggesting that targeting PHGDH may allow to overcome therapeutic resistance [6]. PHGDH has been implicated in resistance mechanisms in various cancers such as melanoma, particularly through metabolic rewiring of serine biosynthesis [7]. However, to our knowledge, this is the first study to elucidate the functional role of PHGDH in anaplastic thyroid cancer (ATC) [8].

The dabrafenib-trametinib combination is the approved standard of care for BRAF V600E-mutant anaplastic thyroid cancer (ATC) [1]. This study was designed as a mechanistic study to dissect the feedback signaling and metabolic rewiring induced by RAF inhibition itself. Therefore, we first investigated resistance-associated changes in dabrafenib monotherapy. This strategy minimizes confounding by MEK blockade and further clarifies the attribution of RAF inhibition effects, facilitating interpretation of the EGFR-PHGDH-stem cell/metabolic axis. Based on these results, we plan to expand the validation of the approved dabrafenib-trametinib combination to include pharmacological and genetic confounding and formal synergistic analysis [9].

We observed therapeutic responses such as a decrease in p-ERK level, inhibition of colony formation, and a decrease in stemness when the PHGDH inhibitor NCT503 was administered alone or in combination with dabrafenib. RNA sequencing (RNA-seq) analysis suggested that PHGDH inhibition affected mitogen-activated protein kinase (MAPK) signaling, metabolic reprogramming, and cell plasticity [10]. In particular, the expression of Leucine-rich repeats and immunoglobulin-like domains 1 (LRIG 1) increased in the resistant state, suggesting that it may act as a negative feedback regulator that suppresses the EGFR–PHGDH axis [11]. The increased expression of LRIG1 was associated with reduced EGFR and pERK signaling [12].

These findings provide a rationale for exploring combination treatment strategies in BRAF V600E mutant thyroid cancer by jointly illuminating the functional crosstalk between the PHGDH-EGFR axis and LRIG1-based suppression of resistance.

## Results

### PHGDH expression is elevated in malignant anaplastic thyroid carcinoma cells

**Fig. 1:**
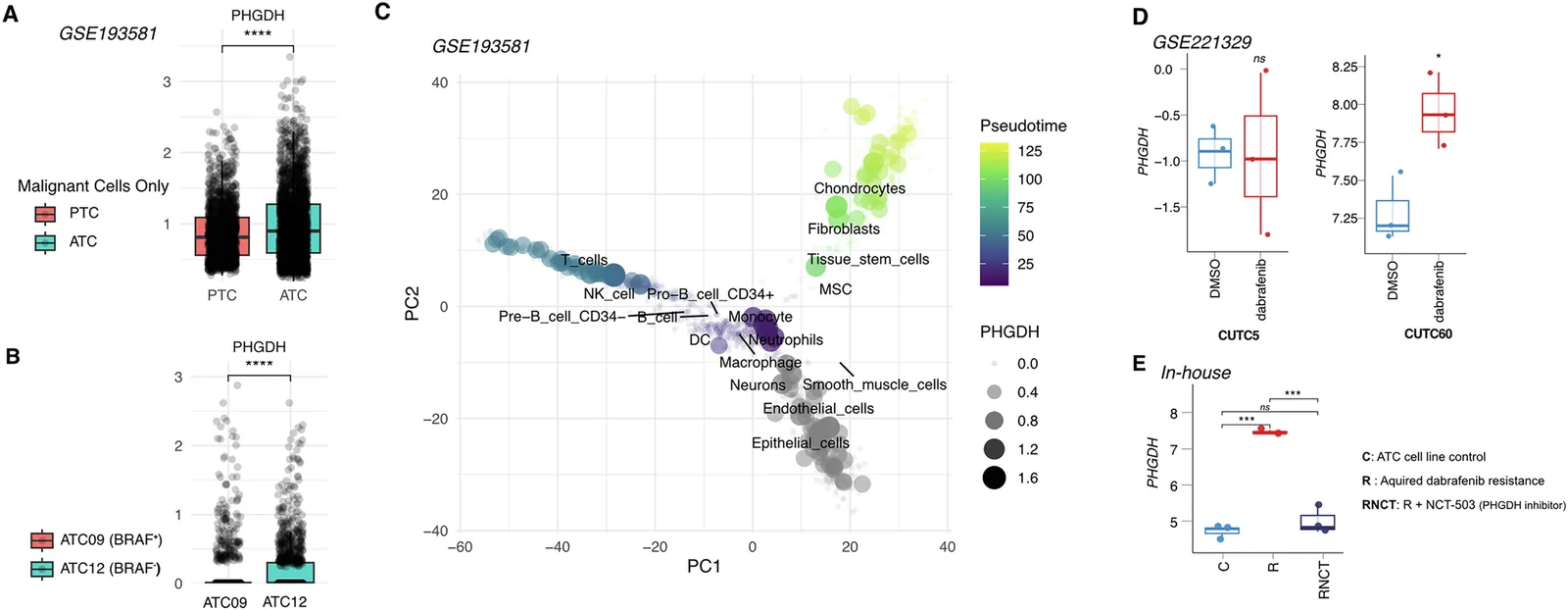
PHGDH expression is elevated in malignant anaplastic thyroid carcinoma cells. **A** PHGDH expression in tumor-cell populations from Papillary Thyroid Carcinoma (PTC) and Anaplastic Thyroid Carcinoma (ATC) in the GSE193581 scRNA-seq dataset. Zero-expression cells were excluded for visualization, whereas statistical analyses included all tumor cells. Basal PHGDH expression was significantly elevated in ATC compared with PTC. Statistical significance was determined using a two-sided Wilcoxon rank-sum test (****p < 0.0001). **B** PHGDH expression in age-, sex-, and stage-matched ATC09 (BRAF V600E-mutant) and ATC12 (BRAF wild-type) tumor-cell populations. Statistical analyses including all tumor cells showed significantly lower PHGDH expression in ATC09, whereas visualization excluding zero-expression cells suggested enrichment of PHGDH-high subpopulations in ATC09, indicating intra-tumoral heterogeneity (two-sided Wilcoxon rank-sum test, ****p < 0.0001). **C** Trajectory analysis of ATC sample (GSE193581) showing PHGDH expression along pseudotime. PCA plot displays the trajectory of immune cell differentiation. Cell clusters are color-coded by cell type, and a pseudotime gradient indicates differentiation progression. Dot size represents the level of PHGDH expression, ranging from low (small, gray) to high (large, black). Dendritic cells (DC) are located centrally, with T cells differentiating outwards, and PHGDH expression generally low with a slight increase in some differentiated cell types. **D** PHGDH expression in PTC and ATC cell lines treated with DMSO or Dabrafenib from the GSE221329 bulk RNA-seq dataset. Box plots show PHGDH expression in PTC (CUTC5) and ATC (CUTC60) cell lines treated with DMSO (control) or dabrafenib for 48 h. PHGDH expression is significantly increased in dabrafenib-treated ATC cells. **E** PHGDH expression in in-house dabrafenib-resistant ATC cells and response to PHGDH inhibition. Box plots show PHGDH expression (relative arbitrary units) in control ATC cell lines (**C**), dabrafenib-acquired resistant ATC cell lines (R), and resistant cells treated with a PHGDH inhibitor (RNCT). Statistical significance was determined by two-sided Wilcoxon rank-sum test (***p < 0.001, ns stands for non-significant). Note the dramatic reduction of PHGDH in resistant cells upon inhibitor treatment.

To gain insights into PHGDH expression in ATC and its association with dabrafenib response, we analyzed publicly available single-cell RNA-seq datasets GSE193581, GSE221329, and in-house bulk RNA-seq data. Initial analysis of all cell types in the GSE193581 dataset showed no significant difference in PHGDH expression between papillary thyroid carcinoma (PTC) and ATC (data not shown) [13]. However, when the analysis was restricted to malignant cells only, we observed a significant upregulation of PHGDH expression in ATC compared to PTC (Fig. 1A). This finding highlights the importance of analyzing tumor-specific cell populations to uncover disease-relevant expression patterns. We also investigated PHGDH expression in a pair of age-, sex-, and stage-matched ATC samples, (ATC09 and ATC12) both from females in their 50 s, T4b,>10% mitochondrial content from the GSE193581 dataset, focusing on their BRAF mutation status. A BRAF V600E mutation was present in ATC09, while ATC12 had wild-type BRAF. Although a visual inspection of the plot with zero-expression cells excluded suggested a higher PHGDH level in ATC09 than in ATC12, statistical analysis that includes all cells revealed a significantly lower in PHGDH expression in ATC09 than in ATC12 (Fig. 1B). This result suggests a complex interplay between BRAF mutation status and PHGDH expression in ATC.

#### PHGDH expression during differentiation in non-malignant cells

Given the reported association between PHGDH expression and stemness, we next explored PHGDH expression patterns during differentiation in non-malignant cells from the ATC samples using trajectory analysis [14]. Analysis of immune cell trajectories from an ATC sample identified three distinct branches of differentiation (Fig. 1C) [15]. PHGDH expression remained generally low across most immune cell types, with no clear increasing or decreasing trend along the pseudo time. However, PHGDH expression tended to be lower in the early differentiation stages along the pseudo time. The trajectory plot revealed dendritic cells at the center, with PHGDH expression tending to increase as cells differentiated further, moving away from the center towards cell types such as T cells (Fig. 1C). This suggests that while its expression does not fluctuate dramatically, PHGDH might have subtle roles in specific differentiation stages of immune cells within the tumor microenvironment.

#### PHGDH upregulation in dabrafenib-resistant ATC and sensitivity to PHGDH inhibition

Given that PHGDH has been implicated in cancer stemness and aggressiveness [15], we hypothesized its involvement in dabrafenib resistance [15]. Analysis of bulk RNA-seq data from GSE221329 revealed that PHGDH expression was significantly higher in an ATC cell line (CUTC60) following dabrafenib treatment for 48 hours than in DMSO control, whereas no significant difference was observed in a PTC cell line (CUTC5) (Fig. 1D). This finding aligns with our expectation that PHGDH plays a role in dabrafenib resistance in ATC. Furthermore, our in-house data for cells with acquired dabrafenib resistance demonstrated that they had significantly higher PHGDH expression than the control cells (Fig. 1E). Crucially, treatment with a NCT503 dramatically reduced PHGDH expression in these resistant cells (Fig. 1E). These results strongly suggest that the PHGDH signaling pathway plays an important role in mediating acquired dabrafenib resistance in ATC, positioning PHGDH as a promising therapeutic target to overcome this resistance.

### Characterization of dabrafenib-resistant ATC cells

**Fig. 2:**
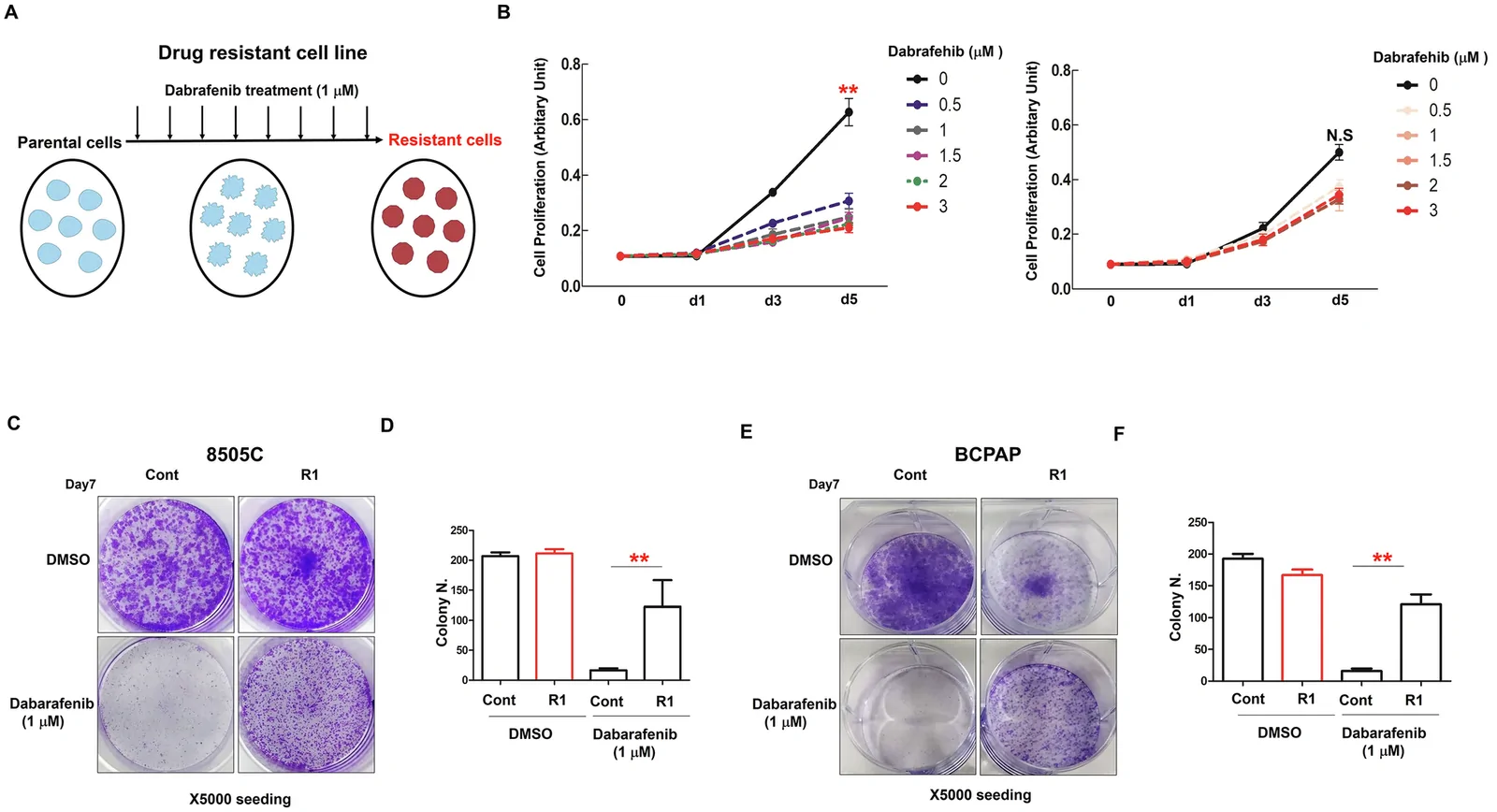
Characteristics of dabrafenib-resistant cell lines. **A** Process of generating Dabrafenib-resistant cell lines from BCPAP and 8505C by treating with Dabrafenib (1 μM). **B** Growth curves according to various concentrations of Dabrafenib in the Dabrafenib-resistant cell line 8505C and the control 8505C. **C**, **D** Colony formation differences after treating 8505C cell line with 1μM Dabrafenib for 7 days were investigated. Figure 2C shows colony formation in 8505 C parental vs. dabrafenib-resistant cells (generated by 4-week low-dose exposure). Note that this is not an acute-response assay, but a comparison of adapted/resistant phenotype. As a result, R1 showed a statistically significant increase in the number of colonies compared to the control group (**p < 0.01). **E**, **F** Colony formation differences after treating BCPAP cell line with 1μM Dabrafenib for 7 days were investigated. As a result, R1 showed a statistically significant increase in the number of colonies compared to the control group (**p < 0.01). All data are presented as the mean ± SD.

To model acquired resistance to dabrafenib, we chronically treated two BRAF V600E-mutant ATC cell lines, 8505C and BCPAP, with dabrafenib for 1 month (Fig. 2A). We then compared the resulting resistant sublines (designated 8505C-R and BCPAP-R) were compared with their parental counterparts to evaluate alterations in proliferative capacity and tumorigenic potential (Fig. 2A–F, Supplementary Fig. 1).

Upon treatment with dabrafenib (0–3 μM) for 5 days, growth of parental cells was inhibited in a dose-dependent manner, with significant reductions at 0.5–3 μM (Fig. 2B, *p* < 0.01). In contrast, growth of 8505C-R cells was not statistically inhibited, confirming the establishment of resistance (Fig. 2B, N.S). Colony formation assays further corroborated this phenotype: dabrafenib significantly reduced the clonogenicity of parental 8505 C cells (Fig. 2C, D; *p* < 0.01), whereas resistant sublines retained high colony-forming ability despite continued drug exposure (Fig. 2E, F, *p* < 0.01 for BCPAP-R).

These findings demonstrate that prolonged dabrafenib exposure induces a stable resistant phenotype in BRAF-mutant ATC cells, accompanied by altered proliferative behavior and enhanced survival [16].

### EGFR signaling is upregulated in dabrafenib-resistant ATC cells and is associated with MAPK pathway reactivation

**Fig. 3:**
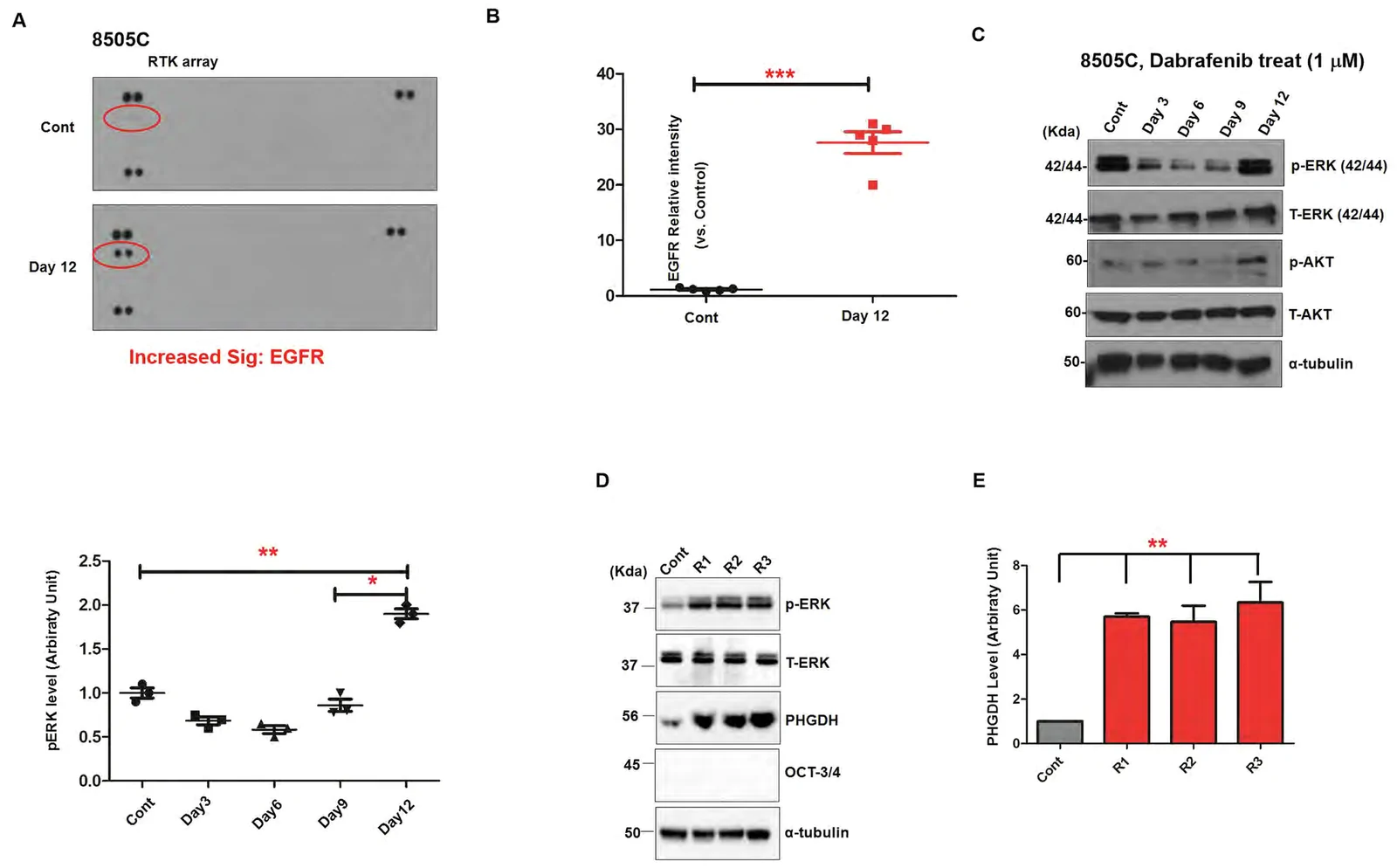
Changes in molecular signaling in dabrafenib-resistant cell lines. **A**, **B** RTK array was analyzed after dabrafenib treatment of 8505 C cells for 12 days. **A** Increase in EGFR signal in the dabrafenib-treated group in comparison with the control group. **B** Quantified of signals shown in (**A**). *** p < 0.001. **C** To determine when the pERK signal in the Dabrafenib-resistant cell line was enhanced, samples treated with Dabrafenib (1 μM) for 0, 3, 6, 9, and 12 days in 8505 C were collected and the changes in pERK signal were investigated. The p-ERK is significantly increased in Day12, and pAKT levels significantly increased in a time-dependent manner. **D**, **E** pERK, PHGDH, and OCT3/4 levels in dabrafenib-resistant cell lines (R1–R3) generated from 8505C. **D** An increase in p-EKR and PHGDH in the dabrafenib-resistant cell line compared to the control. OCT 3/4 was not detected. **E** PHGDH quantification. The data are mean ± SD of three independent experiments (**p < 0.01).

To investigate the mechanisms underlying acquired resistance to dabrafenib, we performed RTK array analysis in BRAF V600E-mutant 8505 C and BCPAP cells treated with or without dabrafenib (Fig. 3A). EGF signaling was significantly upregulated in both 8505C-R and BCPAP-R cells compared to parental controls (Fig. 3B; *p* < 0.001). Time-course analysis of 8505C cells revealed a gradual increase in the levels of ERK and AKT phosphorylation during dabrafenib treatment (Fig. 3C). In dabrafenib-resistant cell lines, increased phospho-EGFR levels were accompanied by elevated pERK levels, indicating MAPK pathway reactivation (Supplementary Fig. 2). Thus, RTK array results indicated an increase in EGFR activation following dabrafenib treatment, which may contribute to MAPK pathway reactivation, rather than complete bypass. Further studies are required to delineate the precise mechanism. Western blot analysis revealed significant PHGDH and pERK up-regulation in dabrafenib-resistant 8505C cells (Figs. 3D, E, Supplementary Fig. 2), consistent with its known role in aggressive tumors. Stemness-related proteins (e.g., OCT4) and epithelial–mesenchymal transition (EMT) markers showed no significant differences in resistant versus control cells (Fig. 3, Supplementary Fig. 3).

These results suggest that dabrafenib-resistant ATC cells bypass BRAF inhibition by upregulating EGFR signaling and reactivating the MAPK pathway, while also engaging PHGDH-driven mitochondrial one-carbon metabolism [17].

### EGF upregulation is not associated with thyroid cancer progression

**Fig. 4:**
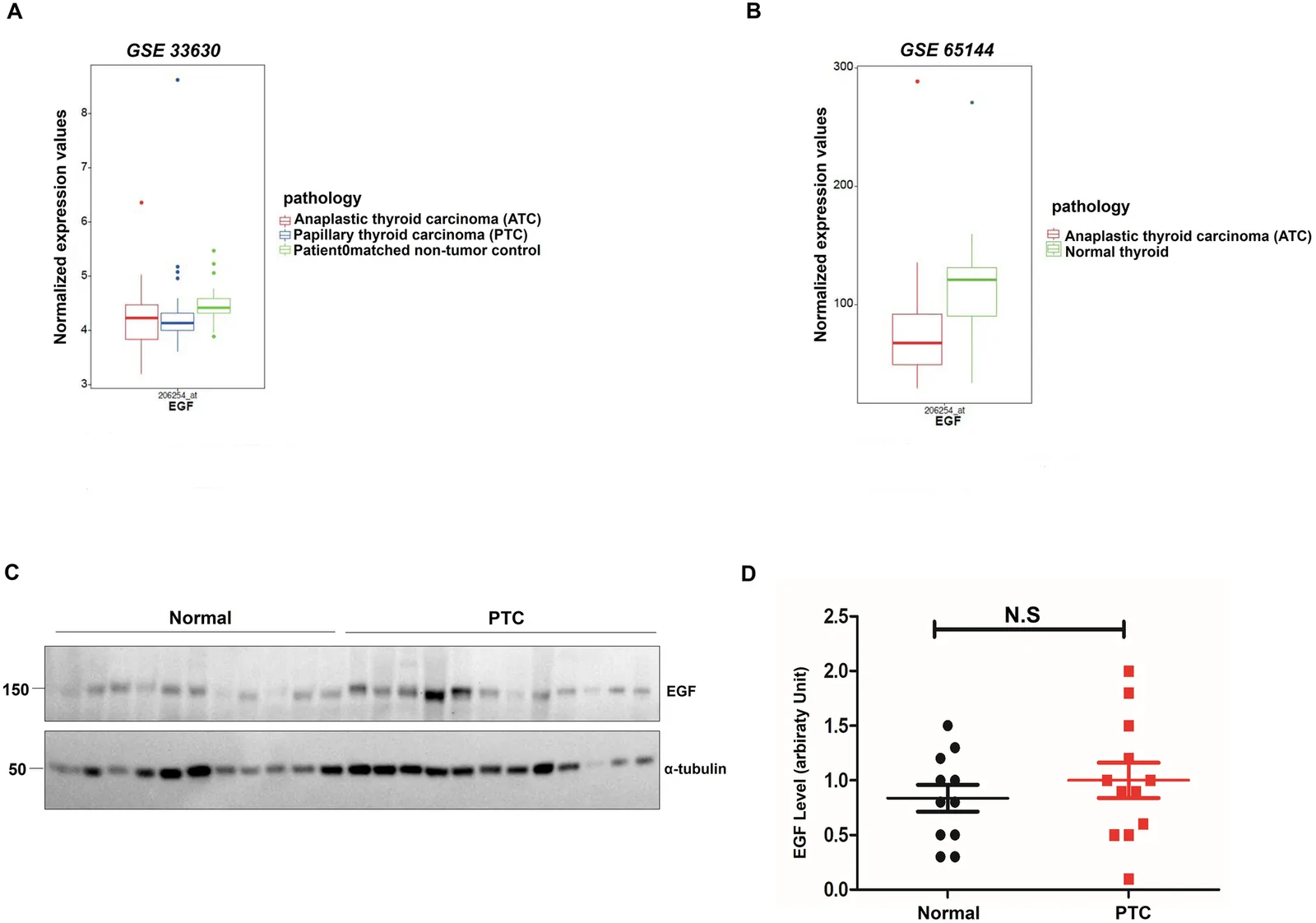
Differences in EGF expression levels between normal and carcinoma (PTC or ATC) human samples. **A** EGF expression levels in normal tissue (patient-matched non-tumor control), anaplastic thyroid carcinoma (ATC), and papillary thyroid carcinoma (PTC). The data are from the GSE33630 dataset, which included a total of 105 samples. There were no significant differences between normal and tumor tissues, although EGF expression was slightly but insignificantly higher in ATC than in PTC. **B** EGF expression levels in normal thyroid tissue and ATC. The data are from the GSE65144 dataset, which included 25 samples. The difference between normal tissue and ATC was not statistically significant. **C** Expression of EGF protein in normal human samples and PTC samples. **D** Quantification of the data shown in (**C**). The data are mean ± SD of three independent experiments. ns not significant.

To determine whether EGF upregulation is associated with thyroid cancer progression regardless of dabrafenib exposure, we analyzed publicly available transcriptomic datasets. In the GSE33630 dataset, EGF transcript levels were compared across 11 ATC, 49 PTC, and 45 non-tumorous thyroid tissues. No significant differences in EGF expression were observed between the groups (Fig. 4A). Similarly, in the GSE65144 dataset (12 ATC and 13 normal samples), EGF expression was slightly higher in normal tissues, with no significant difference (Fig. 4B). To validate these findings, we assessed EGF protein expression in normal and PTC tissues, and found no significant difference (Fig. 4C, D, N.S).

Collectively, these results indicate that EGF upregulation is not associated with thyroid cancer subtype or progression, but instead reflects a response to dabrafenib-induced resistance in BRAF V600E-mutant cells, consistent with our experimental findings.

### PHGDH-associated metabolic reprogramming in dabrafenib-resistant anaplastic thyroid carcinoma

**Fig. 5:**
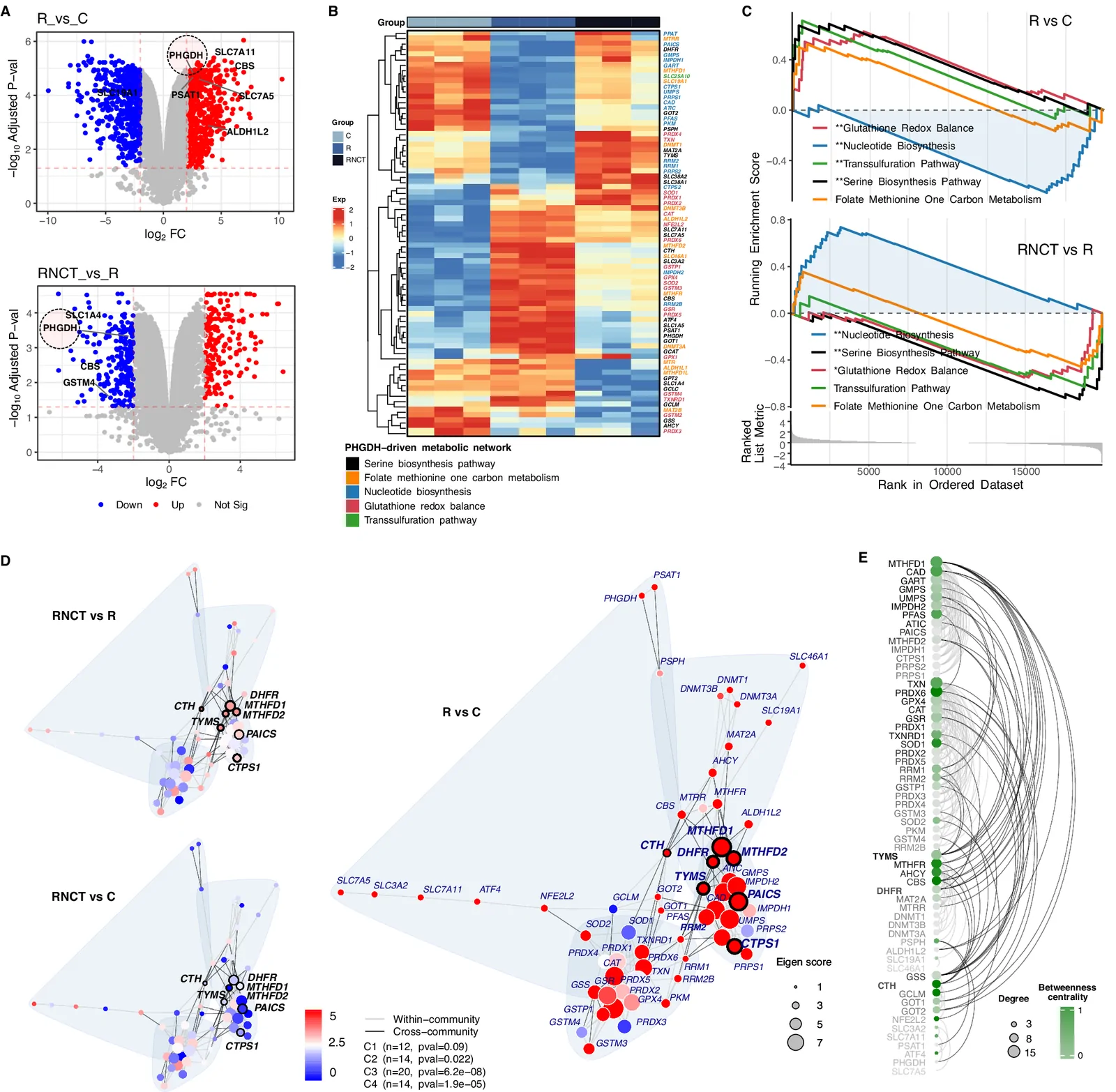
Transcriptional reprogramming of the PHGDH-driven metabolic network in dabrafenib resistance. **A** Volcano plots illustrating differentially expressed genes (DEGs) within the PHGDH-driven metabolic network. Top: Comparison of dabrafenib-resistant (R) vs. control (C) ATC cells. Genes significantly upregulated (log2 FC > 2, FDR < 0.05) are red, downregulated (log2 FC < -2, FDR < 0.05) are blue, and non-significant are gray. Bottom: Comparison of PHGDH-inhibited resistant cells (NCT-503) vs. untreated resistant cells (R). **B** Heatmap showing the expression patterns of genes in the PHGDH-driven metabolic network. The heatmap displays hierarchical clustering of gene expression across control (C), dabrafenib-resistant (R), and PHGDH-inhibitor treated resistant (NCT-503) samples, demonstrating distinct expression patterns in resistance and reversal by PHGDH inhibition. **C** Gene Set Enrichment Analysis (GSEA) plots for key metabolic pathways. Top: Enrichment scores for R vs C comparison. Bottom: Enrichment scores for NCT-503 vs R comparison. Running enrichment scores are shown for “Nucleotide Biosynthesis pathway,” “Glutathione Redox Balance,” “Transsulfuration Pathway,” and “Folate Metabolism One Carbon Metabolism.” Note the inverse enrichment patterns between the two comparisons. **D** Protein–protein interaction (PPI) network plot visualizing the relationships of key metabolic enzymes. Top: R vs C comparison. Bottom: NCT-503 vs C comparison. Nodes represent metabolic enzymes, colored by community (C1–C4) and sized by Eigen score. Edges indicate interactions. Key enzymes like MTHFD1, MTHFD2, DHFR, TYMS, PAICS, CTPS1, and CTH are highlighted. **E** PPI network of the PHGDH-driven metabolic network. Nodes represent proteins, colored by degree centrality (from low/green to high/blue) and sized by between centrality (from small/light to large/dark). Lines indicate predicted protein–protein interactions based on STRING DB. TYMS and CTH showing high betweenness centrality, indicating their pivotal roles as network connectors.

Building upon these findings, we performed a deeper transcriptional analysis focusing on the PHGDH-driven metabolic network, PHGDH-driven which we specifically curated. This network comprises six interconnected pathways: nucleotide biosynthesis, glutathione redox balance, transsulfuration pathway, folate metabolism, one carbon metabolism, and serine metabolism (Supplementary Table 1). These pathways are intrinsically linked to PHGDH activity, as PHGDH initiates the de novo serine biosynthesis pathway. The serine produced then provides crucial one-carbon units that fuel the folate metabolism and one-carbon metabolism, thereby supporting nucleotide biosynthesis. Serine can also be converted to cysteine via the transsulfuration pathway, and cysteine serves as a precursor for glutathione, which is critical for maintaining cellular redox homeostasis. This network collectively forms a comprehensive metabolic axis relevant to cell proliferation and stress response. Volcano plots comparing dabrafenib-resistant cells (R) to control (C) revealed a significant upregulation of genes within the PHGDH-associated metabolic network (FDR < 0.05, fold change > 4), including PHGDH itself (Fig. 5A, top). Conversely, resistant cells treated with a PHGDH inhibitor (R-NCT-503) showed a significant downregulation of genes in comparison with R, indicating successful pathway perturbation (FDR < 0.05, fold change < 4) (Fig. 5A, bottom). A heatmap of these PHGDH-driven metabolic network genes clearly demonstrated distinct and reciprocal expression patterns in the R vs. C and R-NCT-503 vs. R comparisons, underscoring the consistent and widespread activation of this network in resistant cells and its subsequent suppression by PHGDH inhibition (Fig. 5B).

Gene set enrichment analysis (GSEA) further illuminated the metabolic shifts associated with dabrafenib resistance and PHGDH inhibition. Interestingly, the nucleotide biosynthesis pathway was significantly depleted in in R vs. C, while other pathways such as the glutathione redox balance, transsulfuration pathway, and folate metabolism one carbon metabolism were enriched (Fig. 5C, top; Supplementary Table 2). Strikingly, this pattern was precisely reversed in the R-NCT-503 vs. R comparison, where PHGDH inhibition led to a significant enrichment of the nucleotide biosynthesis pathway and a depletion of the other pathways (Fig. 5C, bottom; Supplementary Table 3). This dynamic interplay strongly suggests that the PHGDH-driven metabolic network, particularly its influence on nucleotide biosynthesis, plays a pivotal role in dabrafenib resistance and its circumvention. The regulation of nucleic acid biosynthesis is a well-established mechanism for cancer cells to escape chemotherapy, and our data indicate that this mechanism can be effectively targeted by PHGDH pathway inhibition.

To identify key influential nodes within the PHGDH-driven metabolic network, we constructed a protein–protein interaction (PPI) network by using the String database and integrating expression patterns and the community centrality, degree, and betweenness scores (Fig. 5D, E). Unsupervised network analysis revealed several highly influential genes, including MTHFD1, MTHFD2, DHFR, TYMS, PAICS, CTPS1, and CTH, as core components of this network. MTHFD1, MTHFD2, and DHFR are central enzymes in the one-carbon metabolism pathway and folate cycle, which are crucial for the synthesis of nucleotides, amino acids, and S-adenosylmethionine. Their upregulation in resistant cells highlights the heightened metabolic demand for building blocks necessary for rapid proliferation and adaptation. TYMS (thymidylate Synthase) is a critical enzyme in de novo pyrimidine synthesis, directly involved in DNA replication and repair. Its increased expression underscores the enhanced nucleic acid synthesis in resistant cells. PAICS (phosphoribosyl aminoimidazole carboxylase, phosphoribosyl aminoimidazole succinocarboxamide synthetase) and CTPS1 (CTP synthase 1) are also key enzymes in nucleotide biosynthesis. The high betweenness centrality scores for TYMS and CTH indicate their critical positions within the network as “bridges” or central connectors that facilitate information flow between different parts of the metabolic network. Disrupting these nodes, therefore, could have widespread impacts on the entire network, making them attractive targets for therapeutic intervention.

### PHGDH inhibition reverses changes in lncRNA expression associated with dabrafenib resistance, highlighting potential links to DNA repair

**Fig. 6:**
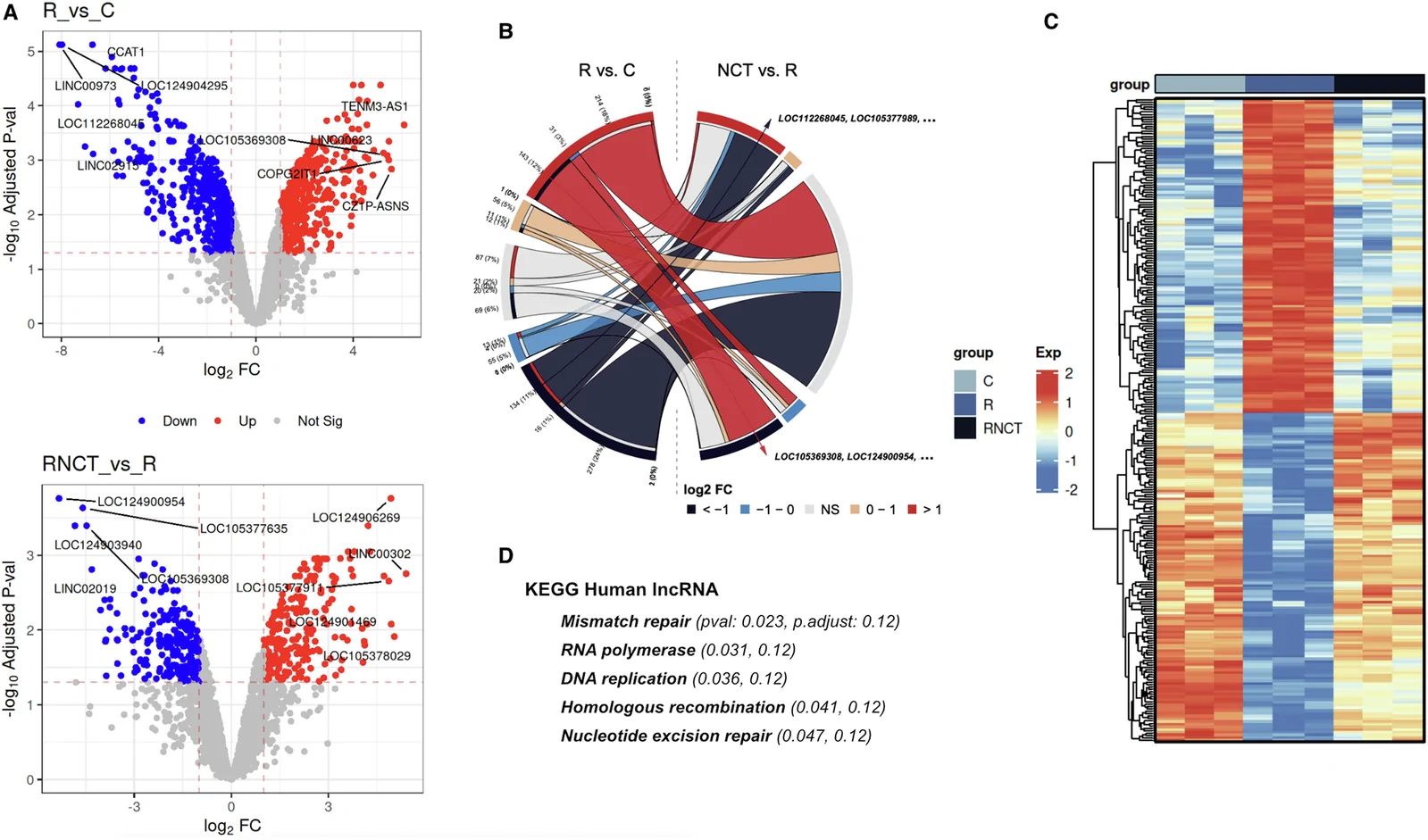
PHGDH inhibition reverses lncRNA expression patterns associated with dabrafenib resistance and enrichment for DNA repair pathways. **A** Volcano plots showing differentially expressed lncRNAs in dabrafenib-resistant (R) vs. control (C) ATC cells (top) and PHGDH-inhibited resistant (NCT-503) vs. untreated resistant (R) ATC cells (bottom). Red dots represent significantly upregulated lncRNAs, blue dots represent significantly downregulated lncRNAs (Adjusted p-val < 0.05), and gray dots represent non-significant lncRNAs. **B** Chord diagram visualizing the log2 fold changes of lncRNAs between the R vs C (outer ring, colored by log2FC) and NCT vs R (inner connections, colored by log2FC in R vs C) comparisons. The diagram illustrates the reversal of expression for many lncRNAs upon PHGDH inhibition. **C** Heatmap displaying the expression patterns of the top differentially expressed lncRNAs across control (**C**), dabrafenib-resistant (R), and PHGDH-inhibitor treated resistant (NCT-503) ATC cells. Expression values are z-scored across rows. **D** Table showing the top-ranking KEGG human lncRNA pathways identified by over-representation enrichment analysis based on the differentially expressed lncRNAs (*p*-val and adjusted *p*-val are indicated).

Profiling the expression patterns of long non-coding RNAs (lncRNAs) revealed a striking trend. A significant proportion of lncRNAs that were either upregulated or downregulated in R cells compared to C cells exhibited a reverse expression pattern in R-NCT-503 cells (Fig. 6A, C). A chord diagram visualizing the log2 fold differences in lncRNAs between the R vs. C and R-NCT-503 vs. R groups revealed a directional reversal of expression for a substantial number of lncRNAs (Fig. 6B). Such a clear reciprocal expression pattern is noteworthy and suggests a strong regulatory link between PHGDH activity and lncRNA expression in the context of acquired resistance. To gain insight into the potential functional implications of this differential lncRNA expression, we performed over-representation enrichment analysis using the KEGG database of human lncRNAs. Notably, the top-ranking pathways enriched for the lncRNAs that showed significant changes in expression in our comparisons were predominantly involved in DNA repair (Fig. 6D, Supplementary Table 4). These included the mismatch repair, RNA polymerase, DNA replication, homologous recombination, and nucleotide excision repair pathways, although these enrichments did not reach statistical significance at a conventional adjusted *p*-value of 0.05. These findings strongly suggest that PHGDH activity influences the expression of lncRNAs that may play a role in DNA repair, hinting at a potential mechanism by which PHGDH inhibition could impact the cellular response to DNA damage or genomic instability associated with drug resistance. Further investigation into the specific roles of these lncRNAs in DNA repair and resistance mechanisms is warranted.

### Potential role of PHGDH in modulating cancer stemness in dabrafenib-resistant ATC

Building upon our previous observation suggesting that PHGDH may play a role in regulating the stemness composition of ATC, we sought to investigate the expression patterns of cancer stemness-associated genes in the context of dabrafenib resistance and PHGDH inhibition [14]. To this end, we compiled an extended set of marker genes related to cancer stem cell identity based on previous studies, including Galiè et al. [18], Hwang et al. [19], and Malta et al. [20]. Additionally, to encompass a broader understanding of stemness, markers indicative of stem cell identity and pluripotency were incorporated from Ramalho-Santos et al. [21] (Supplementary Table 5) [22]. The markers represent genes widely recognized for their roles in defining normal and cancer stem cells, as well as those altered in mesenchymal tumor cells compared to mesenchymal stem cells.

**Fig. 7:**
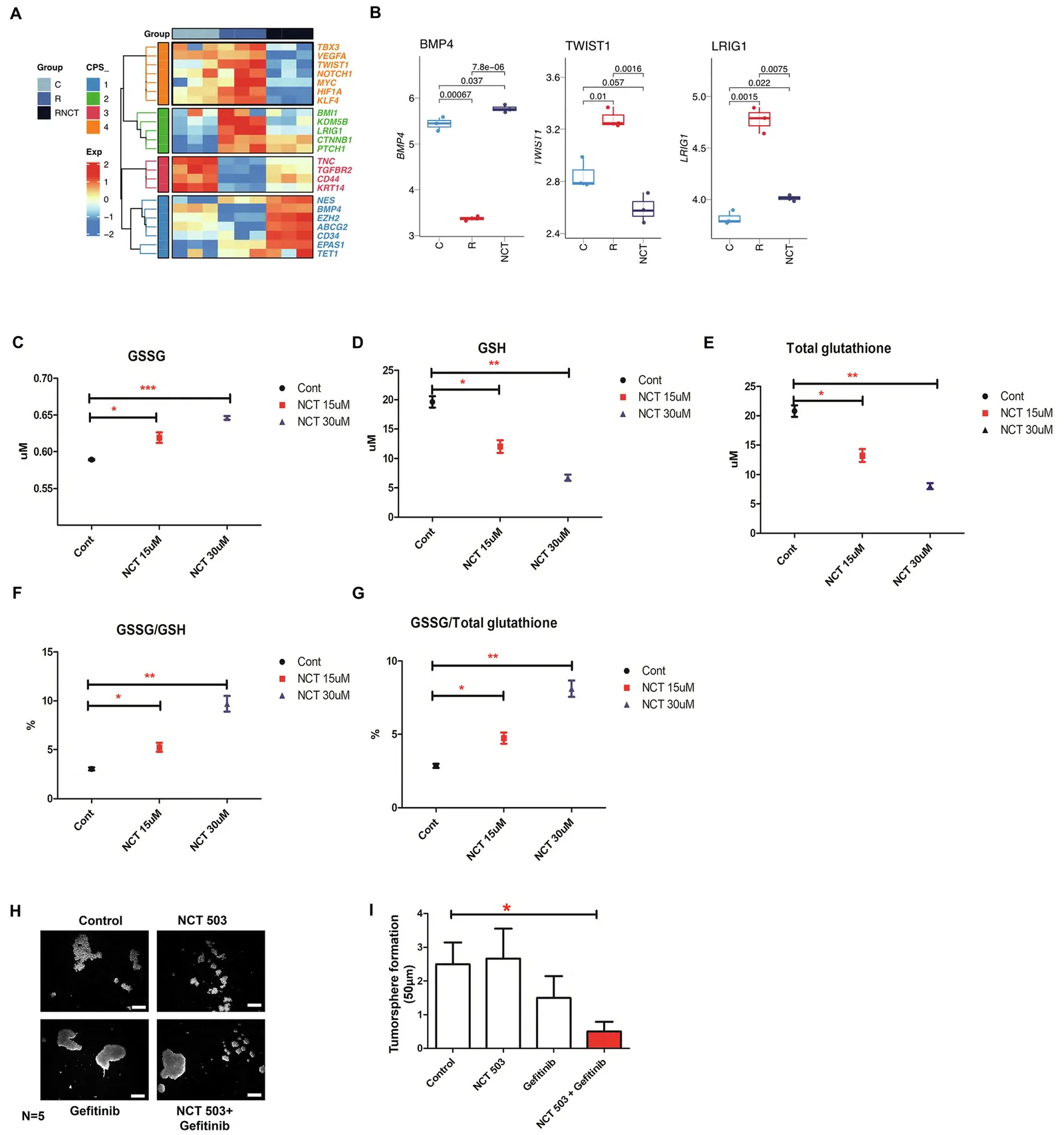
PHGDH and its association with cancer stemness in dabrafenib resistance. **A** Heatmap of curated cancer stemness markers in dabrafenib-resistant ATC cells. The heatmap displays the expression patterns of 40 curated cancer stemness markers across control (C), dabrafenib-resistant (R), and PHGDH-inhibitor treated resistant (NCT-503) ATC cells. Genes were grouped into four distinct expression patterns (Groups 1–4) by consensus clustering. Expression values are z-scored. **B** Differential expression of selected cancer stemness markers. Box plots show the expression levels of BMP4, TWIST1, and LRIG1 in control (C), dabrafenib-resistant (R), and PHGDH-inhibitor-treated resistant (NCT-503) ATC cells. Statistical significance was determined by two-sided *t*-test (*p*-values are indicated above bars). Redox imbalance induced by PHGDH inhibition in dabrafenib-resistant 8505C cells. Quantification of oxidized glutathione (GSSG; **C**), reduced glutathione (GSH; **D**), total glutathione (**E**), GSSG/GSH ratio (**F**), and GSSG/total glutathione ratio (**G**) following treatment with the PHGDH inhibitor NCT-503 (15 μM and 30 μM). PHGDH inhibition increased oxidative stress, as indicated by elevated GSSG levels and increased GSSG/GSH and GSSG/total glutathione ratios, while reduced GSH and total glutathione levels were observed in a dose-dependent manner. **H**, **I** PHGDH inhibition suppresses tumorsphere formation in dabrafenib-resistant ATC cells. Representative images of tumorsphere formation under control, NCT-503, gefitinib, and combined NCT-503 plus gefitinib treatment conditions are shown in (**H**). Quantification of tumorspheres larger than 50 μm is presented in (**I**). Combined treatment with NCT-503 and gefitinib markedly reduced tumorsphere formation compared with single treatments, suggesting that PHGDH contributes to cancer stemness and sphere-forming capacity in dabrafenib-resistant ATC cells. Scale bars, 100 μm. Data are presented as mean ± SD (*p < 0.05). The data are presented as the mean ± SD of three independent experiments (**p < 0.01 and ***p < 0.001).

Using this curated gene set, we performed consensus clustering using our in-house cells. Among the cancer stemness markers, we found four distinct expression patterns (Groups 1–4), indicating their differential transcriptional regulation in response to dabrafenib resistance and PHGDH inhibition (Fig. 7A). Consistent with these findings, similar expression patterns were also evident within our broader stemness marker set (Supplementary Table 4 and Supplementary Fig. 4). Group 1 had high expression in control cells but lower expression in R and R-NCT-503 cells, suggesting potential involvement of these genes in maintaining non-resistant state or their downregulation upon resistance acquisition. Conversely, Groups 2 and 3 had higher expression in resistant than in C cells, with Group 3 showing some reversal in R-NCT-503 cells. Group 4 displayed unique patterns that warrant further investigation.

Notably, within these expression patterns, we observed striking differential expression of specific stemness-related genes. The gene for BMP4 (encoding bone morphogenetic protein 4), a known regulator of cell fate and stem cell maintenance, had significantly higher expression in R cells than in C cells, and its expression appeared to be further increased in R-NCT-503 cells (Fig. 7B). The gene for TWIST1 (Twist family BHLH transcription factor 1), a key inducer of EMT and stemness, was also significantly upregulated in R compared to C cells; interestingly, its expression level was tended to further decrease in R- NCT-503 cells, suggesting a potentially complex involvement in resistance (Fig. 7B). Finally, the gene for LRIG1 (leucine-rich repeat and immunoglobulin-like domain containing Nogo receptor–interacting protein 1), which has been implicated in regulating stem cell self-renewal and tumor suppression, had significantly higher expression in R than in C cells, and it was maintained or slightly reduced in R- NCT-503 cells (Fig. 7B). These clear changes in the expression of genes for BMP4, TWIST1, and LRIG1 suggest that PHGDH- driven metabolic reprogramming may influence specific signaling pathways and molecular programs associated with cancer stemness, thereby potentially contributing to dabrafenib resistance [9]. To further investigate whether PHGDH contributes to the maintenance of cancer stemness through redox regulation, we examined glutathione metabolism following PHGDH inhibition in dabrafenib-resistant 8505 C cells. Quantification of oxidized glutathione (GSSG), reduced glutathione (GSH), and total glutathione revealed that treatment with the PHGDH inhibitor NCT-503 significantly increased intracellular oxidative stress in a dose-dependent manner (Fig. 7C–G). Specifically, GSSG levels and the GSSG/GSH and GSSG/total glutathione ratios were significantly elevated, whereas reduced GSH and total glutathione levels were markedly decreased following NCT-503 treatment. These findings indicate that PHGDH inhibition disrupts cellular redox homeostasis and antioxidant capacity in dabrafenib-resistant ATC cells.

Because redox balance is closely associated with the maintenance of cancer stem-like properties, we next examined whether PHGDH inhibition affects tumorsphere-forming ability. Tumorsphere formation assays demonstrated that combined treatment with NCT-503 and gefitinib markedly suppressed sphere formation compared with either single-agent treatment alone (Fig. 7H, I). In particular, the number of tumorspheres larger than 50 μm was significantly reduced in the combination treatment group, suggesting that simultaneous inhibition of PHGDH and EGFR signaling effectively impairs stemness-associated self-renewal capacity in dabrafenib-resistant ATC cells. Collectively, these findings support the notion that PHGDH-mediated metabolic adaptation contributes to cancer stemness and therapeutic resistance, at least in part through regulation of intracellular redox balance and EGF-associated signaling pathways.

### Dual inhibition of PHGDH and BRAF suppresses tumorigenic potential and stemness in dabrafenib-resistant ATC cells

**Fig. 8:**
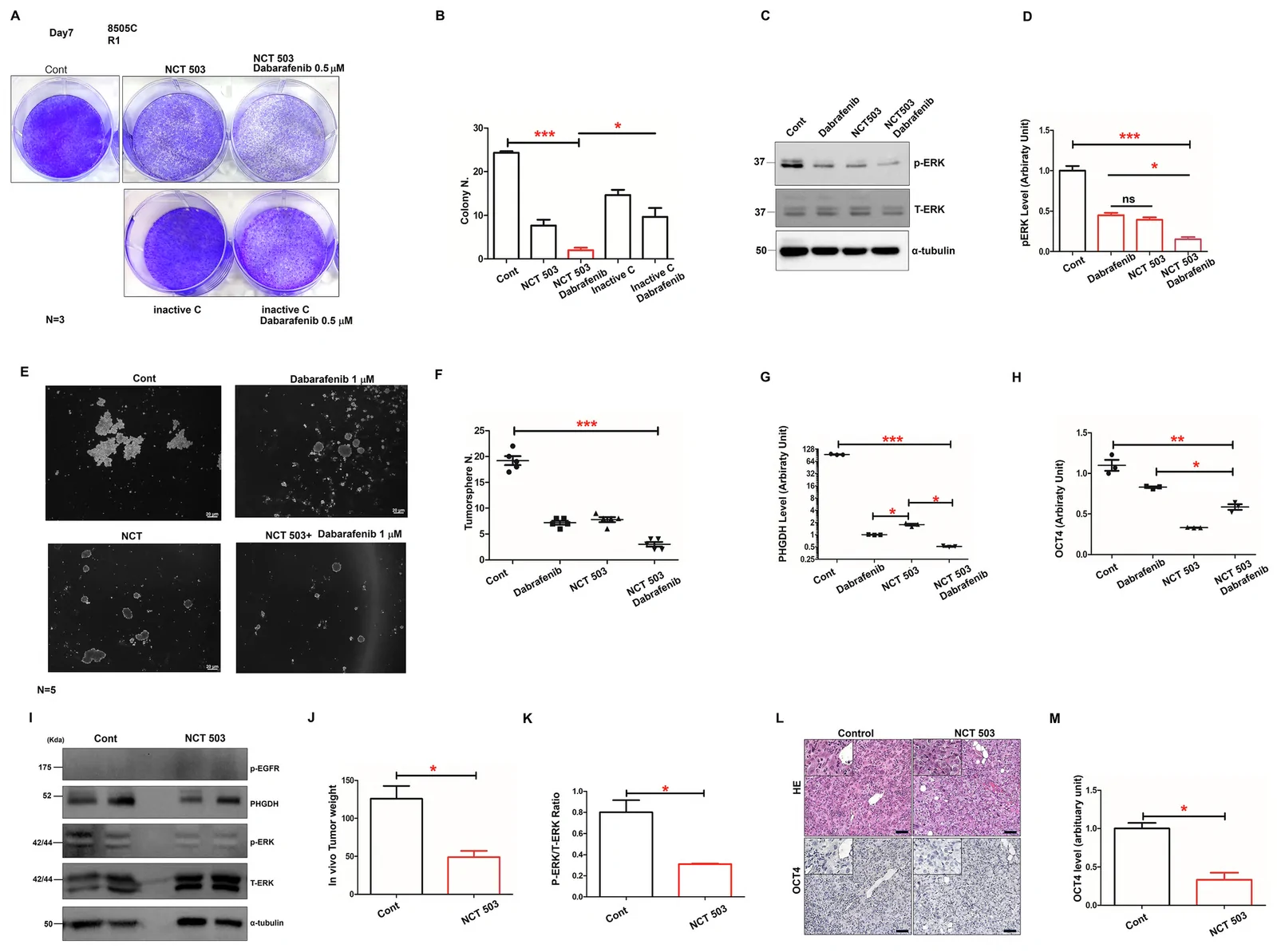
Effect of EGF and PHGDH suppression on colony and tumor sphere formation. **A**–**C** PHGDH and EGF are essential for cell proliferation and tumorigenesis. **A**, **B** Cell proliferation and colony formation in dabrafenib-resistant 8505C cells. **A** Colonies formed on day 7. The number of colonies was reduced by treatment with NCT503 (PHGDH inhibitor, 15 μM) in comparison with the control group, and it was synergistically reduced in NCT503 + dabrafenib treatment. **B** The number of colonies formed by dabrafenib-resistant 8505 C cells on day 7 was significantly reduced by NCT503 + dabrafenib treatment (***p < 0.001). A significant decrease was also observed in the NCT503 only compared to control group (*p < 0.05). **C**, **D** Changes in molecular signaling on day 7. Compared to the control, the pERK signal decreased significantly in the dabrafenib (D), NCT503 (N) and D + N groups. However, the difference in pERK levels between the dabrafenib (D), NCT503 (N) and D + N groups was minimal (*** *p* < 0.001; ns, no statistically significant difference). **E**, **F** Tumor sphere formation in the same groups. **F** Quantification of the number of tumor spheres larger than 50 μm in each group and in 8505 C cells treated with NCT503 (15 μM) + dabrafenib on day 10 (*** *p* < 0.001; ns, no statistically significant difference). **G** After the tumor sphere formation experiment (**E**), the levels of PHGDH mRNA were examined in each sample. NCT503 (15 μM) treatment significantly suppressed PHGDH, and its level slightly recovered in the NCT503 + dabrafenib treatment group (**p < 0.01). **H**, **I** After the tumor sphere formation experiment (**E**), the levels of OCT4 mRNA were examined in each sample. A statistically significant decrease in stemness markers (OCT 4) was observed in the NCT503 + dabrafenib treatment group ((*p < 0.05, **p < 0.01, *** *p* < 0.001). **I**–**K** In vivo validation of PHGDH inhibition in tumor tissues. Western blot analysis of tumor tissues obtained from xenograft models treated with control or NCT503 is shown in (**I**). PHGDH inhibition reduced the levels of p-EGFR and p-ERK, while total ERK levels remained relatively unchanged. Quantification of tumor weight is presented in (**J**), demonstrating a significant reduction in tumor growth following NCT503 treatment. The p-ERK/T-ERK ratio was also significantly decreased in the NCT503-treated group (K), indicating suppression of MAPK signaling in vivo. Data are presented as mean ± SD (*p < 0.05). **L**, **M** Immunohistochemical analysis of OCT4 expression in xenograft tumor tissues. Representative OCT4 immunohistochemical staining images from control and NCT503-treated tumors are shown in (**L**). NCT503 treatment markedly reduced OCT4-positive staining compared with the control group. Quantification of OCT4 expression is presented in (**M**), supporting that PHGDH inhibition suppresses stemness-related features in vivo. Scale bars, 50 μm. Data are presented as mean ± SD (*p < 0.05).

To evaluate the synergistic anti-tumor effects of PHGDH and BRAF inhibition, we conducted colony and spheroid formation assays in 8505 C dabrafenib-resistant cells (Fig. 8).

PHGDH inhibition significantly suppressed colony formation, and this effect was further enhanced by co-treatment with dabrafenib (Fig. 8A, B; *p* < 0.001). At a higher Dabrafenib concentrations (1 μM) combined with NCT 503, colony formation was almost completely abolished (data not shown). These results suggest a synergistic inhibition of tumorigenic potential in resistant cells (Supplementary Fig. 5A). At the same time, pERK levels did not differ significantly between monotherapy and combination therapy (Fig. 8C, D). To further validate these findings, we analyzed spheroid formation after 10 days of 3D culture. The number of large spheroids (>50 μm) was significantly decreased by NCT503 treatment alone and was further decreased under combination treatment (Figs. 8E, F), with all comparisons reaching statistical significance (*p* < 0.01). Interestingly, a statistically significant decrease in PHGDH was observed in the drug-treated group compared to the control group (Fig. 8G). Combination treatment with dabrafenib and NCT503 significantly reduced the mRNA and protein levels of key stemness markers, including OCT4, in resistant cells (Fig. 8I) [14]. But, because the tumorsphere environment is low-attachment and is grown with stem cell-inducing factors such as B27, EGF, FGF, it was difficult to observe a rapid decrease in OCT4 levels in the drug-treated group compared to the control group (Fig. 8I). Collectively, these results suggest that dual inhibition of PHGDH and BRAF can attenuate the expression of pluripotency genes, thereby limiting the tumorigenic potential and stemness of dabrafenib-resistant ATC cells.

### Potential role of PHGDH in cancer stemness and adaptive resistance

To validate the role of PHGDH in adaptive resistance and stemness-associated signaling in vivo, xenograft tumor tissues from control and NCT503-treated groups were analyzed. Western blot analysis demonstrated that PHGDH inhibition reduced p-EGFR and p-ERK signaling in tumor tissues, whereas total ERK levels remained relatively unchanged (Fig. 8I). In addition, tumor growth was significantly suppressed in the NCT503-treated group, as evidenced by reduced tumor weight and decreased p-ERK/T-ERK ratios (Fig. 8J, K). Immunohistochemical analysis further revealed that OCT4-positive staining was markedly decreased following NCT503 treatment (Fig. 8L, M), supporting the notion that PHGDH inhibition suppresses stemness-associated features in vivo [23]. Collectively, these findings suggest that PHGDH contributes to stemness-associated and adaptive resistance phenotypes in dabrafenib-resistant ATC cells beyond its canonical metabolic role. Although PHGDH inhibition partially suppresses adaptive resistance, residual cellular plasticity remains, indicating that simultaneous targeting of metabolic adaptation and oncogenic signaling pathways may be required for more effective therapeutic strategies.

## Discussion

### Overview of BRAF V600E-driven resistance in thyroid cancer

In thyroid cancer, the BRAF V600E mutation is reported mainly in ATC and is found in 20–50% of thyroid cancers [24]. If this mutation is present, the MAPK pathway is activated [25]. Activated MAPK1 (ERK2) promotes the growth and metastasis of cancer cells, and its persistent activation contributes to recurrence even after chemotherapy and surgery [13]. Dabrafenib, which was the focus of this study, is an approved drug and a BRAF kinase inhibitor that suppresses phosphorylation of ERK, which is a MAPK downstream of BRAF [26]. However, dabrafenib is not currently used alone because it causes drug resistance. In this study, we constructed a dabrafenib resistance model and analyzed the molecular signals that change significantly during resistance acquisition, in line with previous reports of RTK or NRAS-mediated bypass signaling and clonal evolution [27].

### Potential involvement of the PHGDH–EGFR axis in resistance

The main finding of this study is that PHGDH-associated serine/glycine metabolic rewiring is linked to dabrafenib resistance (Figs. 1–3). In particular, PHGDH expression was significantly increased in the 8505 C cell line that had acquired dabrafenib resistance (Fig. 2), which was associated with the maintenance of cancer stemness (Figs. 3–8). Selective inhibition of PHGDH decreased colony formation and stem cell marker expression, and simultaneously suppressed ERK signaling (as evidenced by a decrease in p-ERK), suggesting the possibility of overcoming resistance (Figs. 3–7). MAPK reactivation (increased p-ERK) is a major mechanism of dabrafenib resistance, as supported by reports of bypass activation of ERK through RTKs such as EGFR and IGF1R (Figs. 3 and 7) [28]. The p-ERK levels were significantly reduced when PHGDH was inhibited by NCT503 treatment, suggesting a functional link between PHGDH and the EGFR–MAPK axis (Figs. 4 and 8) [15]. In other words, not only the EGFR–MAPK pathway but also the PHGDH–serine/glycine metabolic pathway plays an important role in maintaining dabrafenib resistance, and the interaction between them is an important subject of future research (Figs. 6–8) [29]. Consistent with these findings, additional experiments using trametinib further supported the functional importance of MAPK pathway reactivation in dabrafenib-resistant 8505C cells (Supplementary Fig. 6). Trametinib treatment markedly reduced colony formation compared with dabrafenib alone, and combined dabrafenib plus trametinib treatment further suppressed colony-forming capacity (Supplementary Fig. 6A, B). Western blot analysis additionally demonstrated that p-EGFR and p-ERK levels were significantly decreased following trametinib and combination treatment, whereas total ERK levels remained relatively unchanged (Supplementary Fig. 6C, D). These findings further support that persistent MAPK/ERK signaling is critically involved in adaptive resistance.

Furthermore, combined inhibition of PHGDH and MEK signaling using NCT503 and trametinib effectively suppressed colony formation and ERK activation (Supplementary Fig. 6E–H). Combined treatment reduced colony-forming ability more efficiently than single-agent treatment and further decreased p-ERK levels, indicating that PHGDH-mediated metabolic adaptation functionally cooperates with MAPK signaling during resistance acquisition. Together, these results support the notion that simultaneous targeting of metabolic rewiring and MAPK pathway reactivation may represent a more effective therapeutic strategy for overcoming dabrafenib resistance in ATC cells [30, 31]. Furthermore, genetic rescue experiments provided additional evidence supporting a functional relationship between PHGDH expression and MAPK pathway activity (Supplementary Fig. 7). PHGDH knockdown effectively reduced PHGDH protein expression, whereas re-expression of PHGDH in shPHGDH cells restored PHGDH levels. Importantly, restoration of PHGDH expression significantly recovered ERK phosphorylation, indicating that MAPK pathway activity is at least partially dependent on PHGDH expression. Although these experiments do not fully establish direct mechanistic regulation of EGFR signaling by PHGDH, they provide genetic evidence that modulation of PHGDH expression influences downstream MAPK activation. Collectively, these findings support the existence of a functional PHGDH–EGFR/MAPK resistance axis and suggest that PHGDH-mediated metabolic adaptation contributes to the maintenance of dabrafenib resistance through sustained MAPK signaling.

Previous studies have reported that vemurafenib (PLX4032) induces ERBB3 upregulation as a bypass mechanism of BRAF inhibitor resistance in ATC [20]. However, our study utilized dabrafenib, which has been shown in other cancer types, including melanoma and colorectal cancer, to activate EGFR signaling via feedback regulation [18]. This discrepancy may reflect differences in drug-specific off-target profiles and feedback induction kinetics.

In our hands, dabrafenib treatment consistently increased phospho-EGFR levels in 8505C cells, verified by RTK array and immunoblotting. Cell line identity was validated by DNA fingerprinting as previous out lab reports [14]. Therefore, while ERBB3 may also contribute to resistance in some contexts, our results support a model in which EGFR-driven PHGDH induction contributes to dabrafenib resistance in ATC.

Our previous study demonstrated that dabrafenib alone strongly inhibited colony formation in 8505C cells. We then expanded the study on the established dabrafenibresistant cell line. This model was utilized to elucidate drug-adapted resistance mechanisms [21].

### Therapeutic potential of targeting PHGDH

PHGDH inhibition partially attenuated resistance-associated phenotypes, accompanied by reductions in cancer stemness-associated features and p-ERK signaling. In addition, the PHGDH expression level has the potential to be used as a biomarker in personalized medicine in the future, and may also be useful for screening patients with poor dabrafenib response. Targeting PHGDH may also be therapeutically combined with other metabolism-directed anticancer strategies, including inhibition of folate metabolism and nucleotide biosynthesis pathways, and provides a basis for exploring novel combination treatment strategies [32]. The increase in PHGDH expression identified in this study is closely related to metabolic reprogramming of treatment-resistant thyroid cancer cells, and similar trends have been reported in recent literature [33]. In particular, single-cell transcriptome analysis showed that PHGDH expression gradually increases as thyroid cancer progresses from PTC to ATC, and it is most prominent in the mesenchymal ATC (mATC) subtype [34].

This subtype shows both metabolic plasticity and an aggressive phenotype, suggesting that serine/glycine metabolism, including PHGDH, is involved in the metastasis and dedifferentiation. Although a direct link between PHGDH and stemness has not been established, the results of this study suggest that the correlation between the two is significant [35]. ATC cells are strongly dependent on glutaminolysis, and inhibition of this pathway activates one-carbon metabolism as a compensatory mechanism for maintaining survival [36]. According to previous reports, PHGDH inhibition decreases intracellular glutathione (GSH) levels and increases oxidative stress, thereby enhancing sensitivity to existing anticancer agents such as lenvatinib and sorafenib, and has a synergistic effect with existing anticancer drugs (lenvatinib, sorafenib). These findings emphasize that PHGDH is a key metabolic target for overcoming treatment resistance of solid tumors [37]. Therefore, this study suggests that one-carbon metabolic flow centered on PHGDH plays a key role in the dedifferentiation and resistance acquisition of ATC, and that strategies to inhibit it may improve therapeutic responses [38].

### Clinical implications and future research directions

This study demonstrated that PHGDH is not just a metabolic enzyme, but is involved in various tumor biological functions such as resistance to anticancer agents, cancer stem cell maintenance, and MAPK pathway modulation. Therefore, PHGDH can be considered as a multifunctional target that controls cancer resistance and recurrence [39]. Future studies using patient-derived xenograft (PDX) models and additional genetically validated in vivo systems will be necessary to further clarify the mechanistic and translational significance of the PHGDH–EGFR/MAPK resistance axis.

## Methods and materials

### Cell culture

In this experiment, BCPAP, 8505C, and SW1736 thyroid cancer cell lines were used in this study. BCPAP is derived from PTC with a BRAF mutation, while 8505C and SW1736 originate from anaplastic thyroid carcinoma (ATC) with the same mutation. Cells were cultured at 37 °C in RPMI 1640 medium (Gibco, Grand Island, NY, USA) supplemented with 10% fetal bovine serum and 5% CO₂. Subcultures were performed every 2–3 days, and cells were passaged upon reaching 70–80% confluency. Cell line authentication was confirmed by short tandem repeat (STR) profiling [14] and tested for mycoplasma contamination.

### Knockdown of PHGDH by small hairpin RNA

We generated lentiviral vectors expressing small hairpin RNA (shRNA) in the BCPAP cell line. shRNA transfection was performed using two different sequences. The target sequence for the human PHGDH gene was CTTAGCAAAGAGGAGCTGATA (TRCN0000221861, shPHGDH #1). For PHGDH knockdown experiments, BCPAP cells were infected with shRNA-expressing lentiviruses at known titers with a multiplicity of infection (MOD) of 2.5–5. Cell lines were then established by selection with G418.

### Colony formation and cell proliferation assay

Cells were seeded onto 6-well plates at a density of 5 × 10³ cells per well. After 6 days of incubation, non-adherent cells were removed by washing twice with PBS. Attached cells were fixed in 500 µl of 10% formalin for 20 min, followed by staining with 500 µl of 0.1% crystal violet for 20 minutes. Plates were gently rinsed with water. Colonies exceeding a defined size threshold were counted at five random fields per well, and the results were statistically analyzed to compare differences between groups.

To assess cell viability, an MTT [3-(4,5-dimethylthiazol-2-yl)-2,5-diphenyltetrazolium bromide] assay was performed on BCPAP and 8505 C cells treated with dabrafenib. MTT reagent was added to each well and incubated at 37 °C for 4 h. The reaction was terminated by adding 100 µl of detergent solution, followed by a 2-hour incubation at room temperature in the dark. Absorbance was measured at 540 nm using a microplate reader.

### Spheroid formation assay

Tumor sphere formation was assessed using ultra-low attachment 96- and 6-well plates. 8505 C cells were seeded at a density of 3 × 10⁴ cells per well (n = 6). The culture medium consisted of RPMI 1640 supplemented with recombinant human EGF, human FGF, B27 supplement, and 1% penicillin/streptomycin (all from Invitrogen, Thermo Fisher Scientific, Waltham, MA, USA). Fresh medium was added every 3 days, and cells were monitored for 10 days. On day 10, spheres ≥50 µm were counted in five minutes under a light microscope at 100× magnification. Samples were collected for subsequent analysis, including real-time PCR [14].

#### Knockdown of PHGDH by small hairpin RNA

PHGDH knockdown was performed as previously described in our thyroid cancer study. Briefly, 8505 C cells were infected with lentiviral vectors expressing PHGDH-targeting shRNAs (TRCN0000221861, shPHGDH #1 and TRCN0000221862, shPHGDH #2; Thermo Fisher Scientific) in the presence of polybrene, followed by overnight incubation.

### mRNA expression analysis

Quantitative real-time PCR (qRT-PCR) was performed to measure mRNA levels of PHGDH, Oct-4 (octamer-binding transcription factor 4), and NANOG (Nanog homeobox) in dabrafenib-resistant cell lines cultured under stemness-inducing conditions for 10 days. Total RNA was extracted using TRIzol® reagent (Invitrogen, Thermo Fisher Scientific, Waltham, MA, USA), and cDNA was synthesized using the RevertAid First Strand cDNA Synthesis Kit (Fermentas, Thermo Fisher Scientific, Waltham, MA, USA). Each 25 µl PCR reaction contained 1.0 µl cDNA, 12.5 µl QuantiFast SYBR Green PCR Master Mix, 1.0 µl RNase-free water, and 10 pM of each primer. PCR was conducted on a 7500 Fast Real-Time PCR System (Applied Biosystems, Foster City, CA, USA). 18S rRNA served as the internal control. Primer sequences were previously described by Jeon et al. [26] and used as referenced [14]. Thermal cycling conditions were as follows: initial denaturation at 95 °C for 10 s, followed by 40 cycles of 60 °C for 30 s.

### Western blot analysis

Proteins (20–30 μg) from cell lysates and human samples were separated by SDS-PAGE using NuPAGE gels (Invitrogen, Thermo Fisher Scientific, Waltham, MA, USA) and transferred to 0.45 μm nitrocellulose membranes (Amersham Bioscience, Piscataway, NJ, USA). Membranes were blocked with Super Blot blocking buffer (Invitrogen) for 10 minutes, followed by incubation with primary antibodies overnight at 4 °C. After washing three times with TBS-T, membranes were incubated with HRP-conjugated secondary antibodies (1:5000) for 1 h. Detection was performed using enhanced chemiluminescence (Enzo Life Sciences, New York, USA).

Primary antibodies used were as follows: pEGF Receptor (Tyr1068), EGF Receptor, p-ERK (Thr202/Tyr204), ERK, Vimentin, N-cadherin, E-cadherin, pAKT (Ser473), AKT, LRIG1 (Cell Signaling Technology, Cat# 2236, 2748, 4903, 6964, 9102, 9101, 5741, 13116, 3195, 9271, 9272, 12752), PHGDH (Abcam, ab57030), and OCT3/4 (Santa Cruz Biotechnology, sc-5279, 1:200). Protein levels were normalized to β-actin (Cell Signaling Technology, 4970). All experiments were independently performed in triplicate.

### Phospho-receptor tyrosine kinase (RTK) analysis

Phospho-receptor tyrosine kinase (RTK) array analysis was conducted using dabrafenib-resistant 8505 C, BCPAP, SW1736, and corresponding control cell lines. Approximately 300 µg of total protein lysate was incubated with the Human Phospho-RTK Array Kit (R&D Systems, Minneapolis, MN, USA), following the manufacturer’s protocol. Signal intensity was detected by enhanced chemiluminescence (ECL), as used in western blot analysis.

### Pharmacological inhibitors

Dabrafenib, trametinib, and gefitinib were used to generate drug-resistant cell lines. Dabrafenib (Selleck Chemicals, Houston, TX, USA) was dissolved in 100% DMSO (Sigma-Aldrich, St. Louis, MO, USA). An equivalent concentration of DMSO was used as a vehicle control in all experiments. The PHGDH inhibitor NCT 503 and its inactive analog were obtained from Sigma-Aldrich (St. Louis, MO, USA).

#### Measurement of intracellular glutathione levels

Intracellular glutathione levels were measured to assess cellular redox status using the GSSG/GSH Quantification Kit (Dojindo, Cat. No. G257) according to the manufacturer’s instructions. Briefly, cells were treated with NCT-503 at the indicated concentrations, harvested, and counted. A total of 1 × 10^7^ cells per condition were used for the assay. Levels of oxidized glutathione (GSSG), reduced glutathione (GSH), and total glutathione were measured, and the GSSG/GSH ratio and GSSG/total glutathione ratio were calculated to evaluate oxidative redox balance. Data are presented as the mean ± SEM.

#### IHC analysis

The degree of PHGDH and OCT4 protein expression was evaluated by IHC staining using an anti-PHGDH antibody (ab57030; Abcam, sc-5279; Santa Cruz Biotechnology). IHC staining was performed on in vivo mouse samples according to our laboratory protocol. Briefly, paraffin-embedded tissue sections were deparaffinized in xylene and rehydrated through graded ethanol. Antigen retrieval was carried out using citrate buffer, followed by blocking of endogenous peroxidase activity with 3% hydrogen peroxide. After blocking with normal serum, sections were incubated with primary antibodies at 4 °C overnight. Subsequently, biotinylated secondary antibodies were applied, and signals were developed using DAB solution. Sections were counterstained with hematoxylin, dehydrated, and mounted for analysis.

#### In vivo mouse xenograft study

All animal procedures and protocols implemented in this study were approved by the Institutional Animal Care and Use Committee of Konkuk University (Approval No. KU25017), Seoul, Korea. Four-week-old female athymic BALB/c nude mice (Orient Bio, Korea) were used. Sample sizes were determined based on our previous experience with this xenograft model and were consistent with those commonly used in similar studies. Dabrafenib-resistant 8505 C cells were prepared, and 5 × 10⁶ cells in 200 μL of suspension mixed with Matrigel (BD Biosciences, San Jose, CA) were subcutaneously inoculated into the right flank of the mice. Eighteen mice were randomly assigned to two groups (vehicle control and NCT503 treatment). No animals or samples were excluded from the analysis, and no pre-established exclusion criteria were applied. Investigators were not blinded during group allocation, treatment administration, or outcome assessment. Tumor size was measured every two days using calipers, and tumor volume was calculated as length × width² / 2. When the tumor volume reached approximately 100 mm³, treatment was initiated with either vehicle control (DMSO) or NCT503 (4 mg/kg) administered intraperitoneally (i.p.) once daily for 10 days. Body weight was measured daily during the treatment period. At the study endpoint, tumors were excised and weighed. Tumor tissues were divided into two portions: one portion was fixed for immunohistochemical (IHC) staining of PHGDH using anti-PHGDH antibodies (1:100; Abcam, Cambridge, UK) and anti-OCT4 antibodies (1:100; sc-5279; Santa Cruz Biotechnology), and the other portion was used for Western blot analysis.

### RNA sequencing

In vitro thyroid cancer model (8505C cell line). To investigate transcriptomic changes associated with drug resistance, total RNA was extracted from 8505 C thyroid cancer cells under three conditions: untreated control, dabrafenib-resistant, and dabrafenib-resistant cells treated with the PHGDH inhibitor NCT 503. Bulk RNA sequencing was performed to assess differential gene expression. Notably, PHGDH expression was markedly elevated in resistant cells and reduced upon NCT 503 treatment, suggesting its potential involvement in serine metabolism–mediated resistance mechanisms.

### Data acquisition and processing

Raw count data were imported into a DGEList object using the edgeR package in R. Genes with low expression levels were filtered out to improve data quality, and normalization was performed using the trimmed mean of M-values (TMM) method via the calcNormFactors function to account for library size differences. The normalized count data were then transformed to log2-counts per million (logCPM) values using the voom function from the limma package, which models the mean-variance relationship and generates precision weights for each observation [40]. Differential expression analysis was conducted using linear modeling and empirical Bayes moderation, with differentially expressed genes identified at a false discovery rate (FDR) threshold of 0.05. Single-cell RNA sequencing data were processed using the Seurat package in R [41]. Quality control filtering excluded cells with <200 or >5000 detected features and >10% mitochondrial gene expression. After QC, normalization and variance stabilization were performed using the SCTransform workflow, which applies regularized negative binomial regression–based normalization while accounting for technical noise. Cell type annotation was conducted using SingleR with the Human Primary Cell Atlas reference dataset from the celldex package. Differential gene expression analysis between sample groups was performed using FindMarkers (), both on total cells and malignant cells only. Trajectory analysis was performed using Slingshot on PCA coordinates to infer developmental trajectories and pseudotime ordering [42]. Standard statistical thresholds and multiple testing corrections were applied throughout the analysis. Gene expression datasets GSE193581 and GSE221329 were downloaded from the Gene Expression Omnibus (GEO) database. Single-cell RNA-seq data were processed using Seurat (v4.0) in R.

### Unsupervised machine learning

We employed unsupervised machine learning approaches to identify novel gene modules associated with PHGDH metabolic networks and stemness-related pathways. Soft clustering analysis was performed using fuzzy c-means clustering via the means function from the e1071 package, which allows genes to belong to multiple clusters with varying membership degrees, better capturing the complex regulatory relationships in biological networks. Consensus clustering was implemented using R ConsensusClusterPlus package to ensure robust and stable cluster identification across multiple iterations and subsampling procedures. This approach provided confidence measures for cluster assignments and optimal cluster number determination. To identify key influential nodes and analyze their topological significance within the PHGDH-driven metabolic network, a protein-protein interaction (PPI) network was constructed. This network was derived from the STRING database, with associations filtered to include only those with a medium confidence score. The resulting network was then represented and analyzed using the graph package in R. Community detection within this PPI network was performed utilizing the leading eigenvector community detection algorithm. This approach identifies densely connected groups of nodes that likely represent functional modules. The modularity of these identified communities was also assessed. Influential nodes were subsequently identified by evaluating various network centrality measures. Eigenvector centrality was employed to determine node influence based on connections to other well-connected nodes, and this metric was used to scale node sizes in visualizations. Additionally, degree centrality (number of direct connections) and betweenness centrality (extent to which a node serves as a bridge between network parts) were calculated. Nodes exhibiting high scores in these centrality measures, particularly those demonstrating substantial differences in Eigen scores between resistant and PHGDH-inhibitor-treated conditions, were pinpointed as critical influential factors within the network.

### Functional enrichment analysis

To elucidate the biological significance of identified gene sets and modules, functional enrichment analyses were performed. Gene Set Enrichment Analysis (GSEA) was utilized to determine whether predefined sets of genes were significantly overrepresented at the extremes of a ranked gene list [43].

Genes were ranked based on a specified statistic. The GSEA algorithm calculates an enrichment score (ES) by traversing the ranked list, reflecting the enrichment of a gene set. To assess statistical significance, a Normalized Enrichment Score (NES) was calculated by normalizing the ES against a null distribution generated by permutation testing. Nominal p-values were subsequently adjusted for multiple hypothesis testing using the false discovery rate (FDR) method. Over-representation analysis (ORA), based on the hypergeometric test, was additionally employed to identify gene sets that were significantly enriched among differentially expressed genes. This method assesses whether the observed overlap between a list of genes of interest and a predefined gene set is greater than what would be expected by chance. Adjusted p-values, typically using FDR correction, were used to account for multiple comparisons.

### Co-expression network analysis and hub gene identification

To identify key genes with differentially co-expression network in dabrafenib resistance and upon PHGDH inhibition, signed scale-free co-expression networks were constructed. Gene expression correlations were calculated to generate correlation matrices. The eigenvalues and eigenvectors of these correlation matrices were then computed using the eigen function. The absolute difference between the first eigenvectors (representing Eigen scores) of the two conditions was calculated. Genes exhibiting large delta. Eigen values were identified as influential hub genes whose network connectivity and importance significantly shifted between conditions.

### Statistics

Categorical variables were expressed as numbers and percentages, while continuous variables were reported as means ± standard deviations (SDs) or medians with interquartile ranges. Comparisons of continuous variables were performed using Student’s t-test, and categorical variables were compared using Fisher’s exact test. One-way ANOVA was used to assess differences among three or more groups. All Pavlus were two-sided, with *P* < 0.05 considered statistically significant. Graphs were generated using GraphPad Prism version 5.01 (GraphPad Software Inc., San Diego, CA, USA). Data were assessed for normal distribution before applying parametric statistical tests.

## Supplementary information

Supplementary Tables

Full-length uncropped western blot images.

Legends for all supplementary figures

Supplementary Figure 1

Supplementary Figure 2

Supplementary Figure 3

Supplementary Figure 4

Supplementary Figure 5

Supplementary Figure 6

Supplementary Figure 7

## Data Availability

The datasets generated and/or analyzed during the current study are available in the Gene Expression Omnibus (GEO) repository under accession numbers GSE193581, GSE221329, GSE33630, and GSE52144. Additional data supporting the findings of this study are available from the corresponding author upon reasonable request.
