# Supplementary material for: PHGDH inhibition overcomes dabrafenib resistance through metabolic rewiring in BRAF V600E anaplastic thyroid carcinoma
Source: Cell Death Discov. 2026 Aug 15;12:452. doi: 10.1038/s41420-026-03293-7 (PMC13755417; doi:10.1038/s41420-026-03293-7)
Supplement: Supplementary file 1 [file 41420_2026_3293_MOESM10_ESM.pdf]

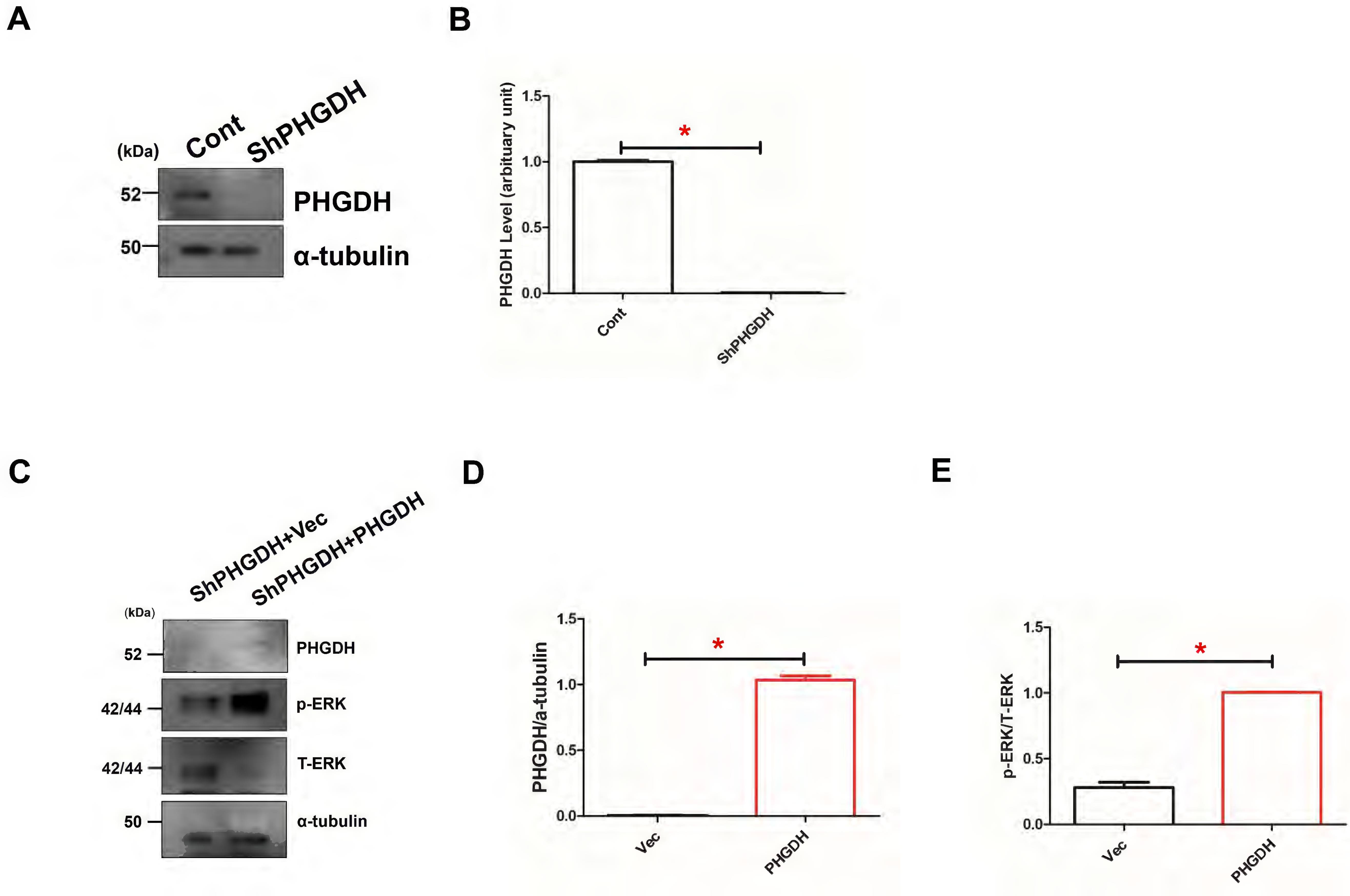

Rescue Experiment
