# Supplementary material for: PHGDH inhibition overcomes dabrafenib resistance through metabolic rewiring in BRAF V600E anaplastic thyroid carcinoma
Source: Cell Death Discov. 2026 Aug 15;12:452. doi: 10.1038/s41420-026-03293-7 (PMC13755417; doi:10.1038/s41420-026-03293-7)

# Figure 3

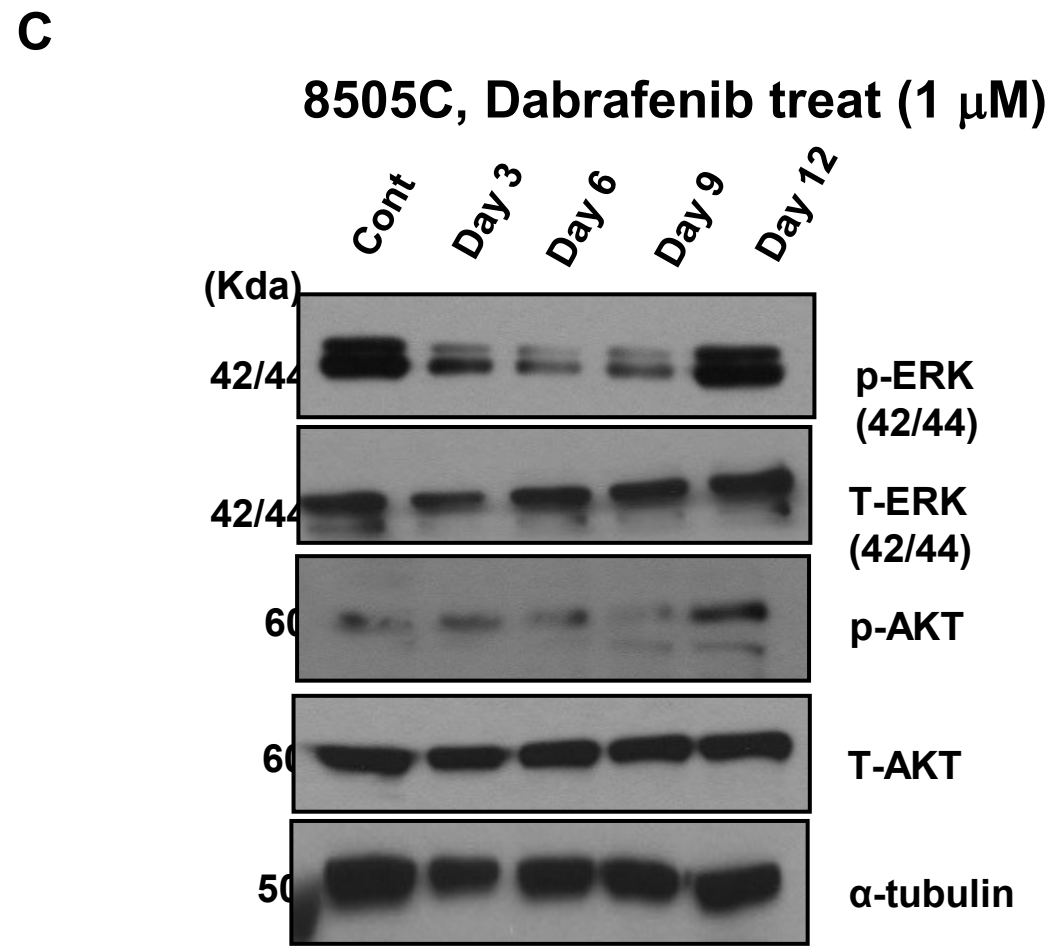
C
8505C, Dabrafenib treat (1 μM)
Cont
Day 3
Day 6
Day 9
Day 12
(Kda)
42/44
42/44
60
60
50
p-ERK (42/44)
T-ERK (42/44)
p-AKT
T-AKT
α-tubulin

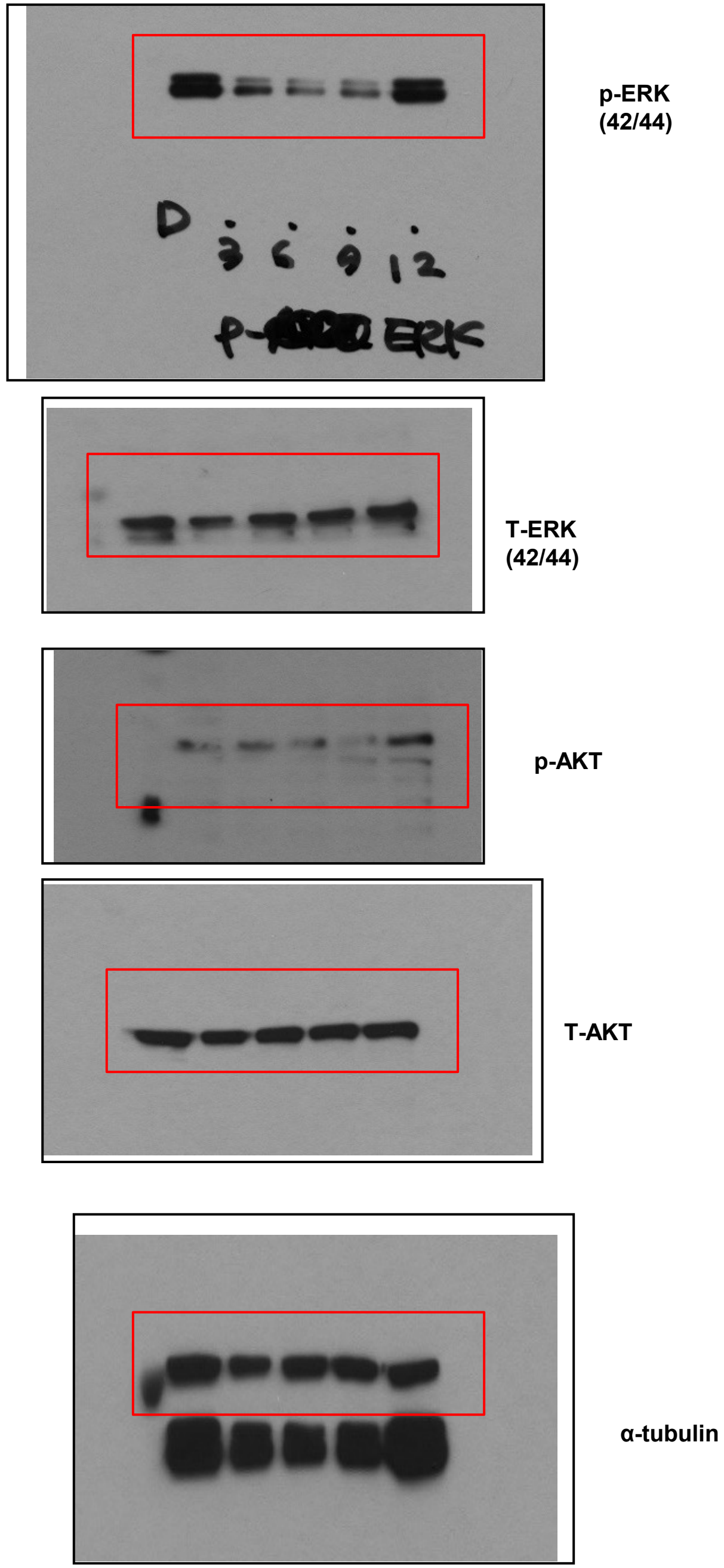
p-ERK (42/44)
T-ERK (42/44)
p-AKT
T-AKT
α-tubulin

# Figure 3

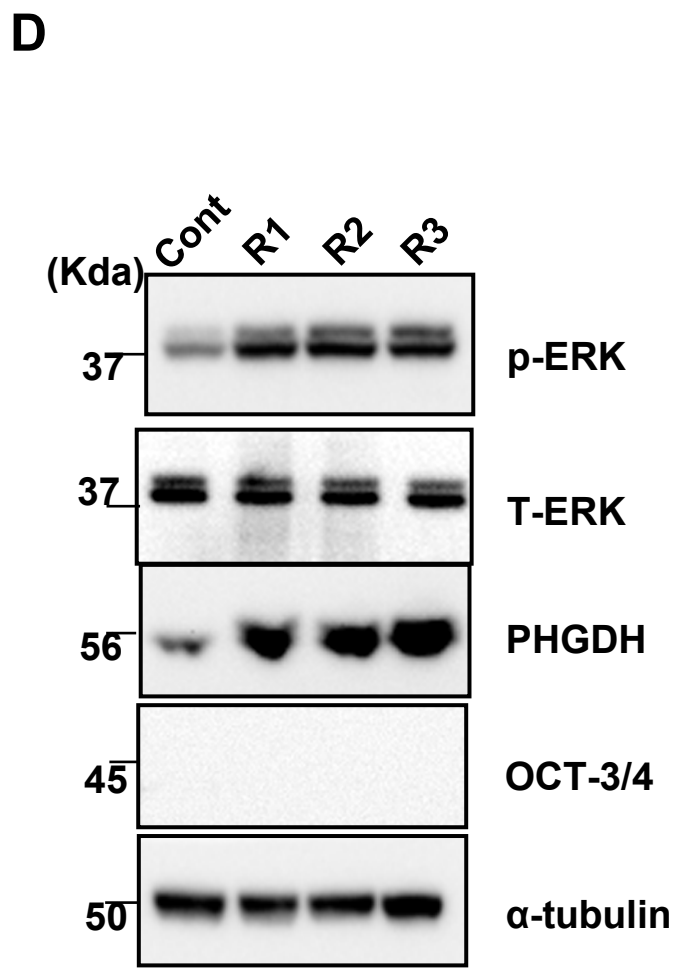
D
(Kda)
Cont
R1
R2
R3
37
p-ERK
37
T-ERK
56
PHGDH
45
OCT-3/4
50
α-tubulin

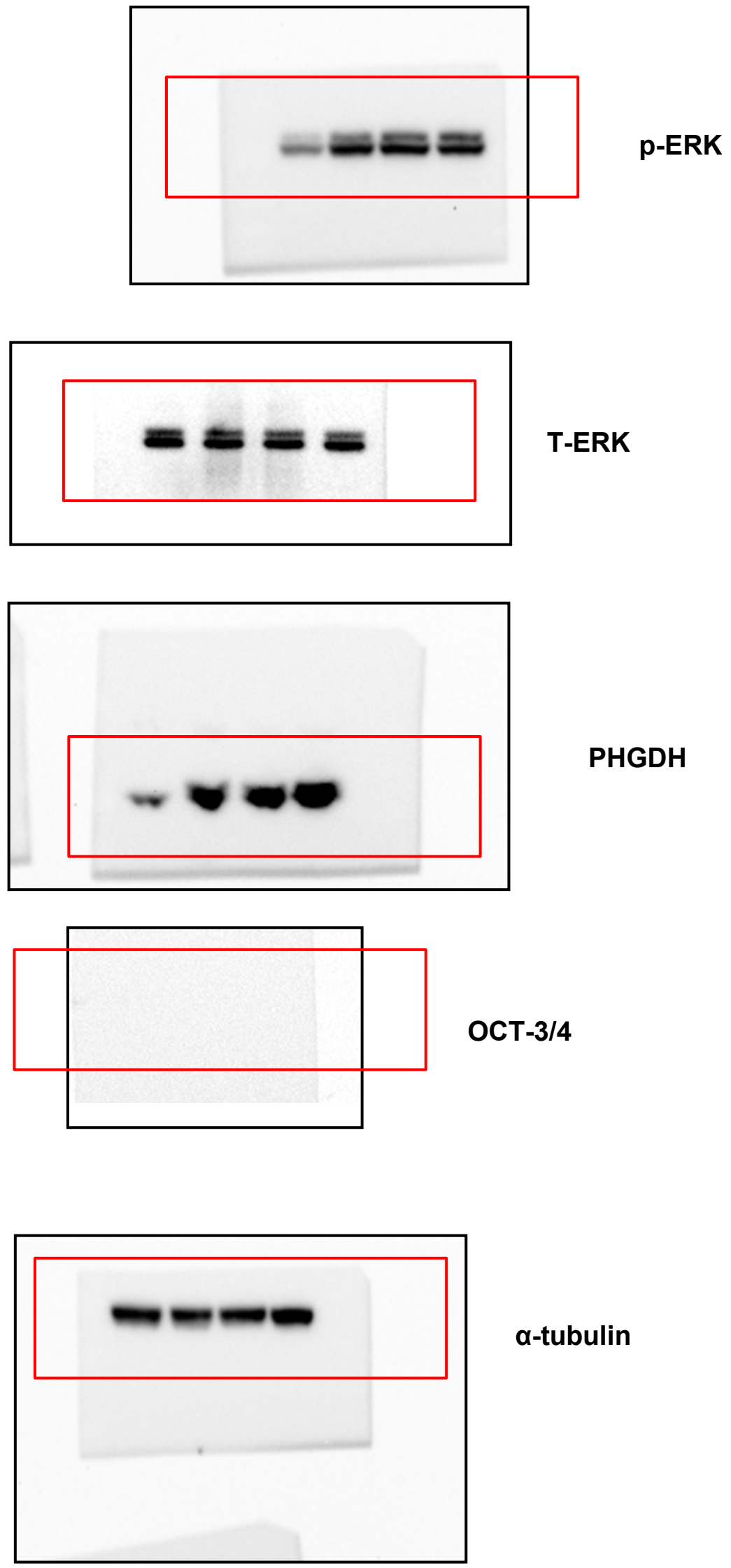
p-ERK
T-ERK
PHGDH
OCT-3/4
α-tubulin

# Figure 4

C

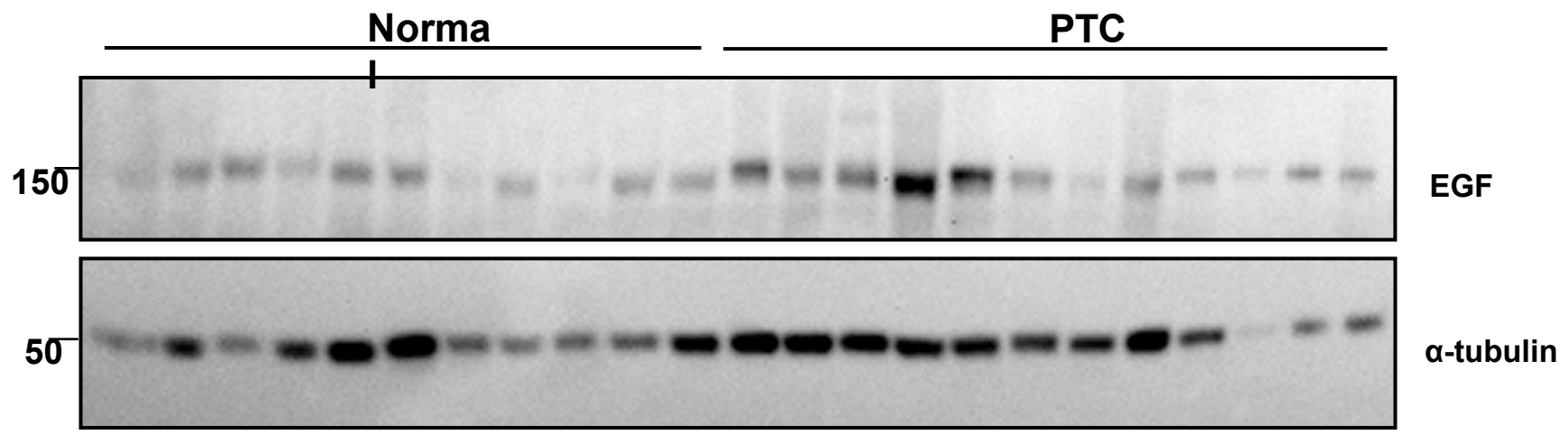


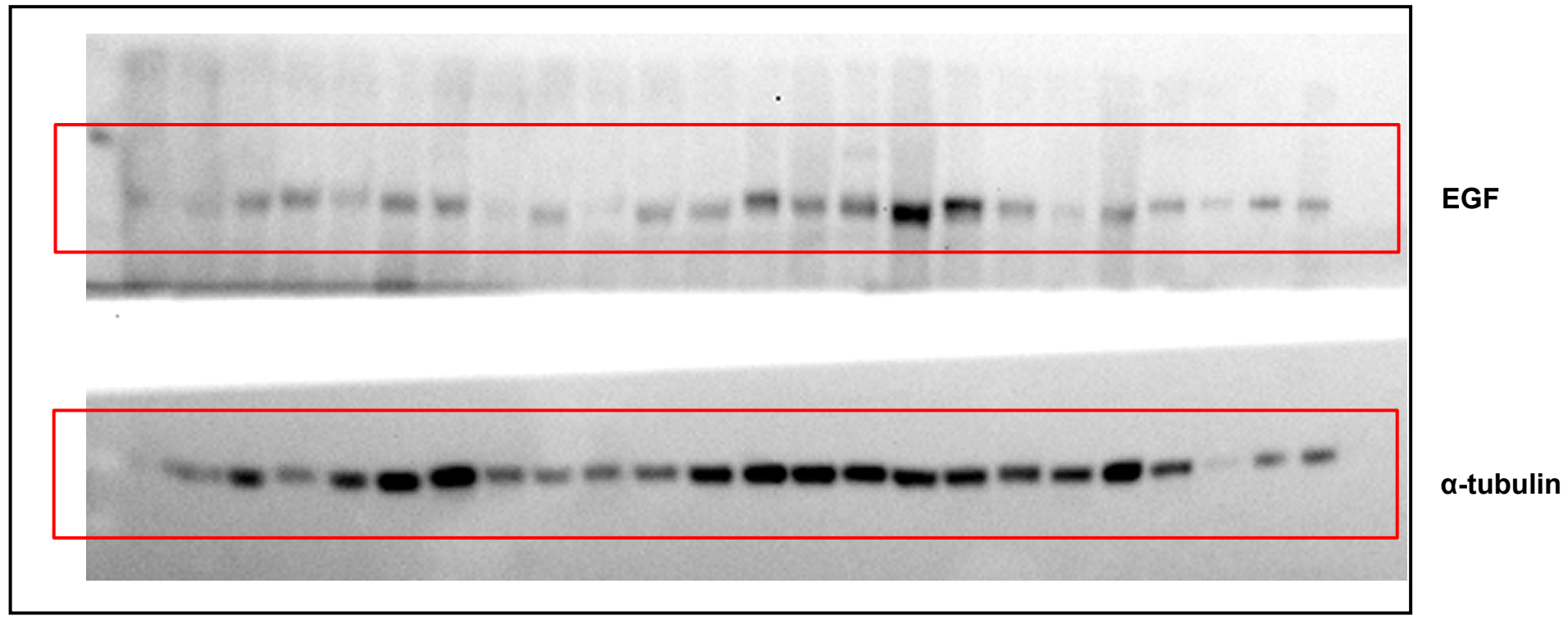

# Figure 8

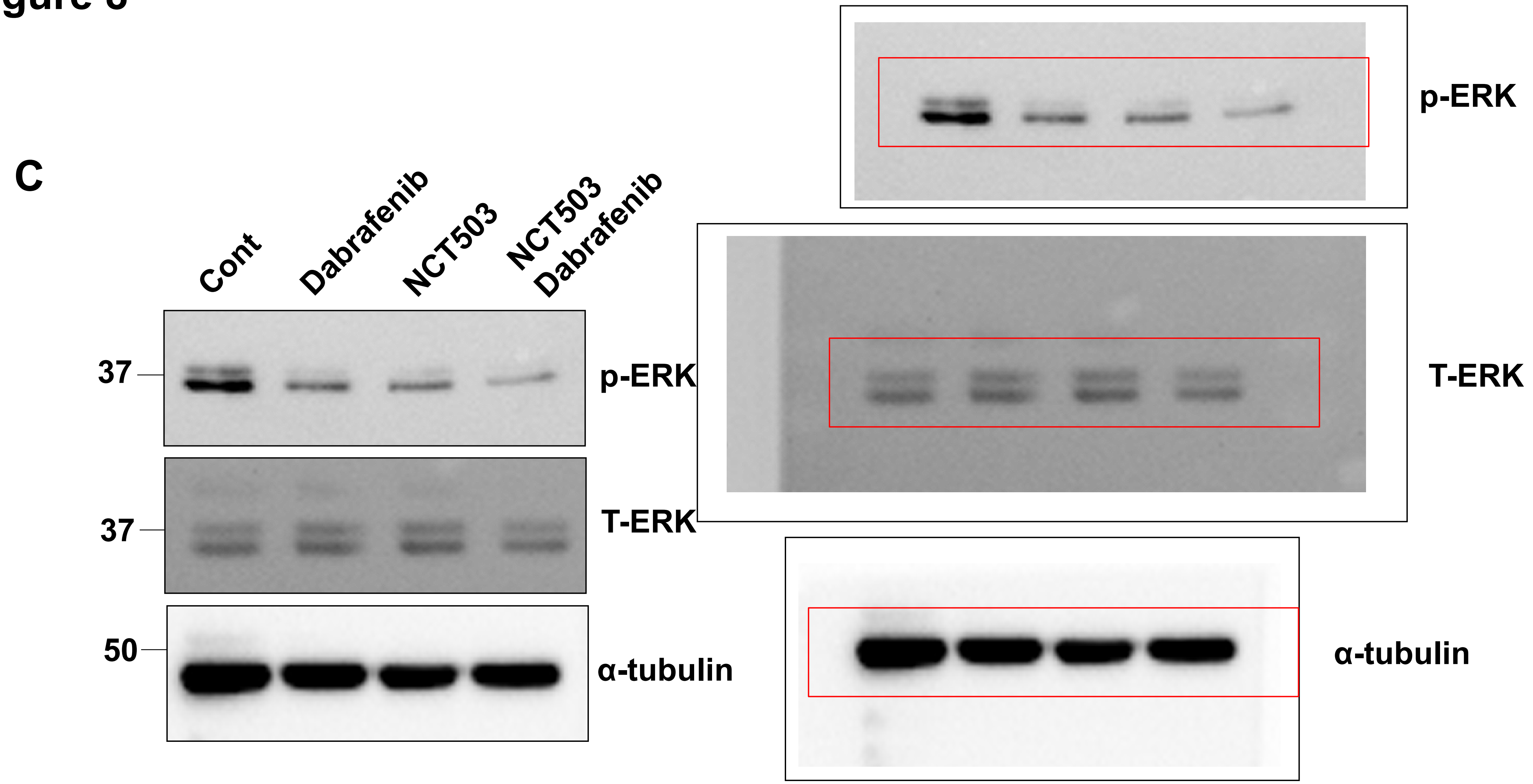
C
Cont
Dabrafenib
NCT503
NCT503
Dabrafenib
37
p-ERK
37
T-ERK
50
α-tubulin
p-ERK
T-ERK
α-tubulin

# Figure 8

I

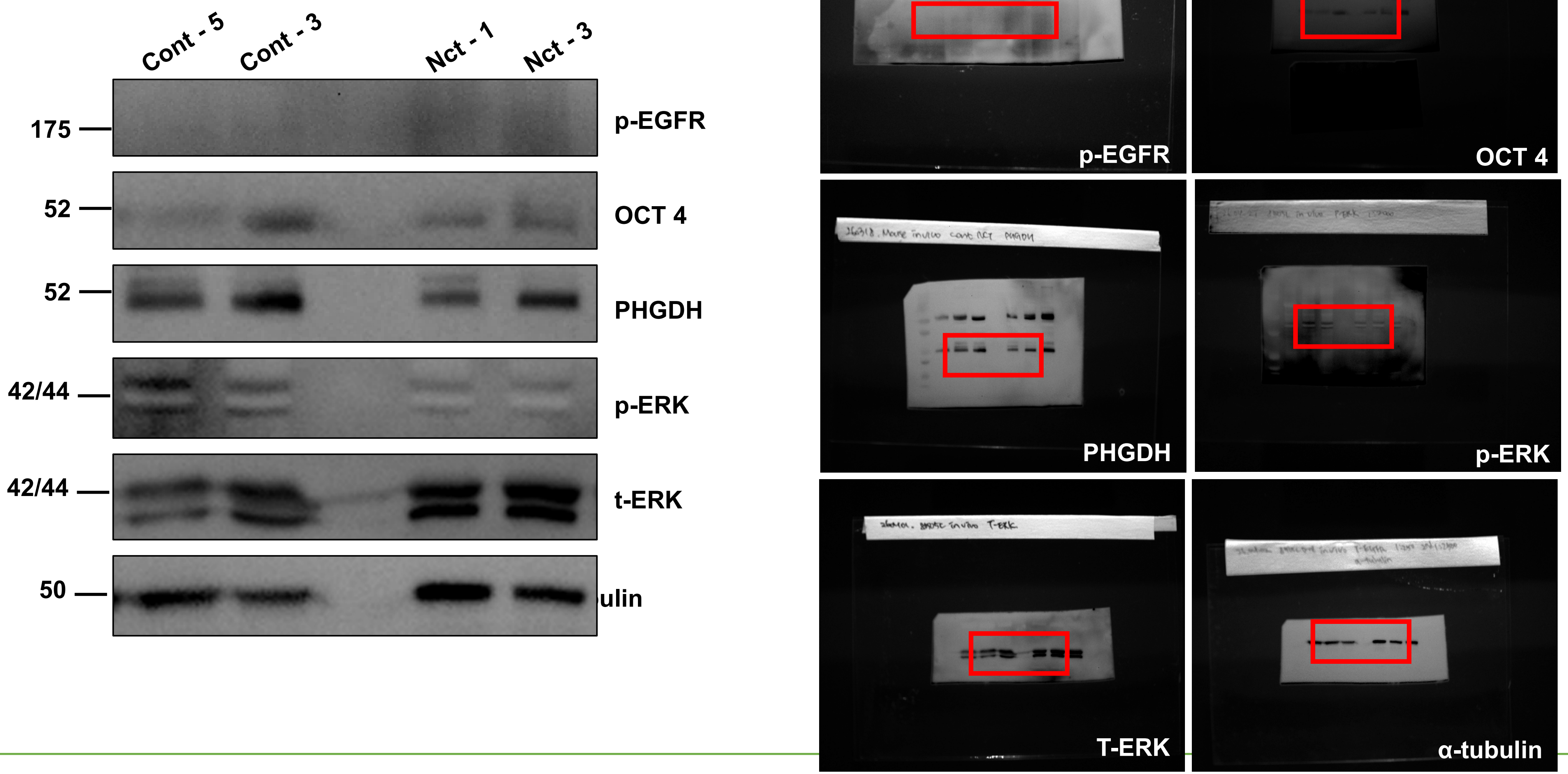

Cont - 5
Cont - 3
Nct - 1
Nct - 3
175
52
52
42/44
42/44
50
p-EGFR
OCT 4
PHGDH
p-ERK
t-ERK
ulin
p-EGFR
OCT 4
PHGDH
p-ERK
T-ERK
α-tubulin

# Supple Figure 6

## western blot_NCT 503, Gefi

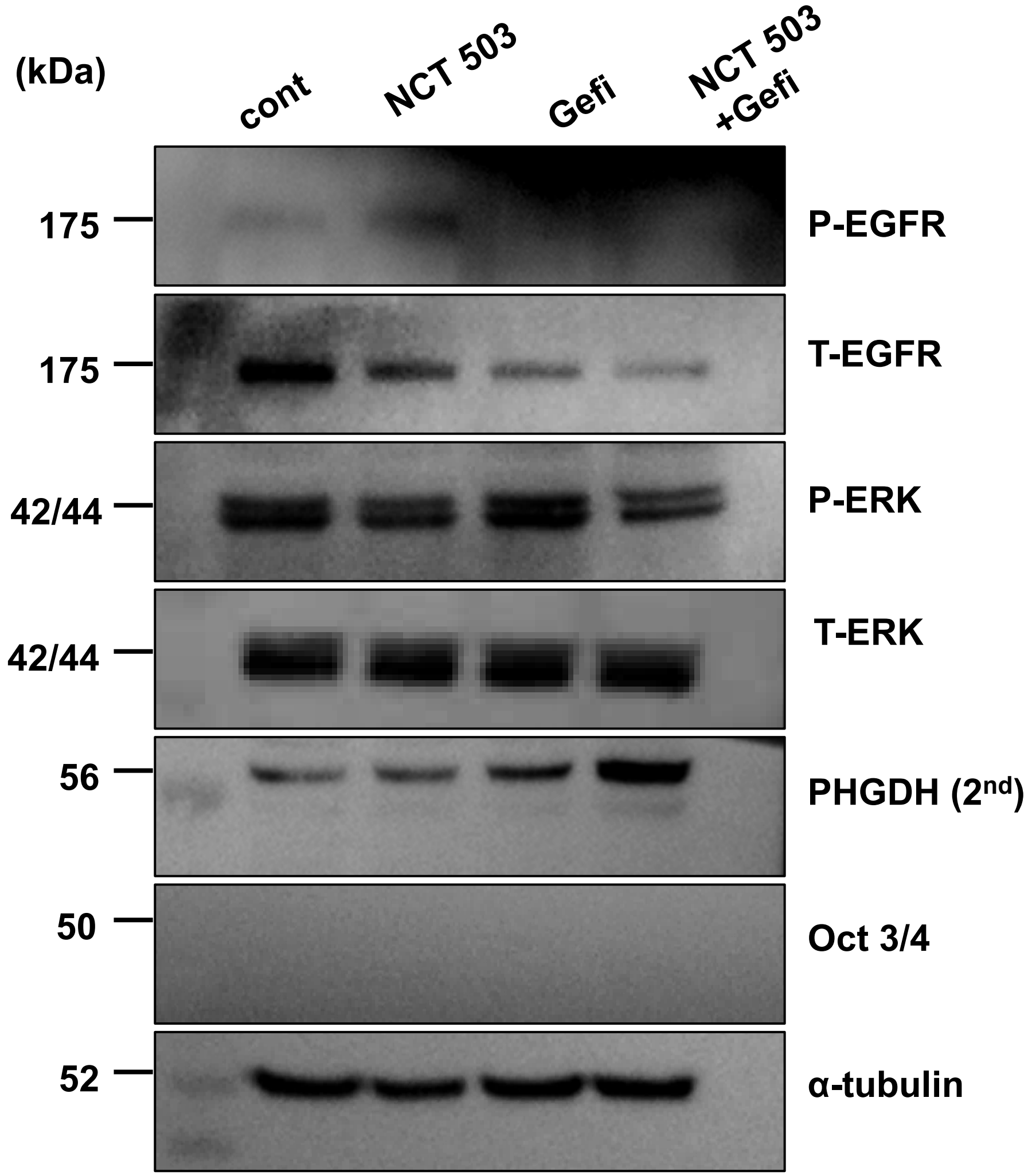


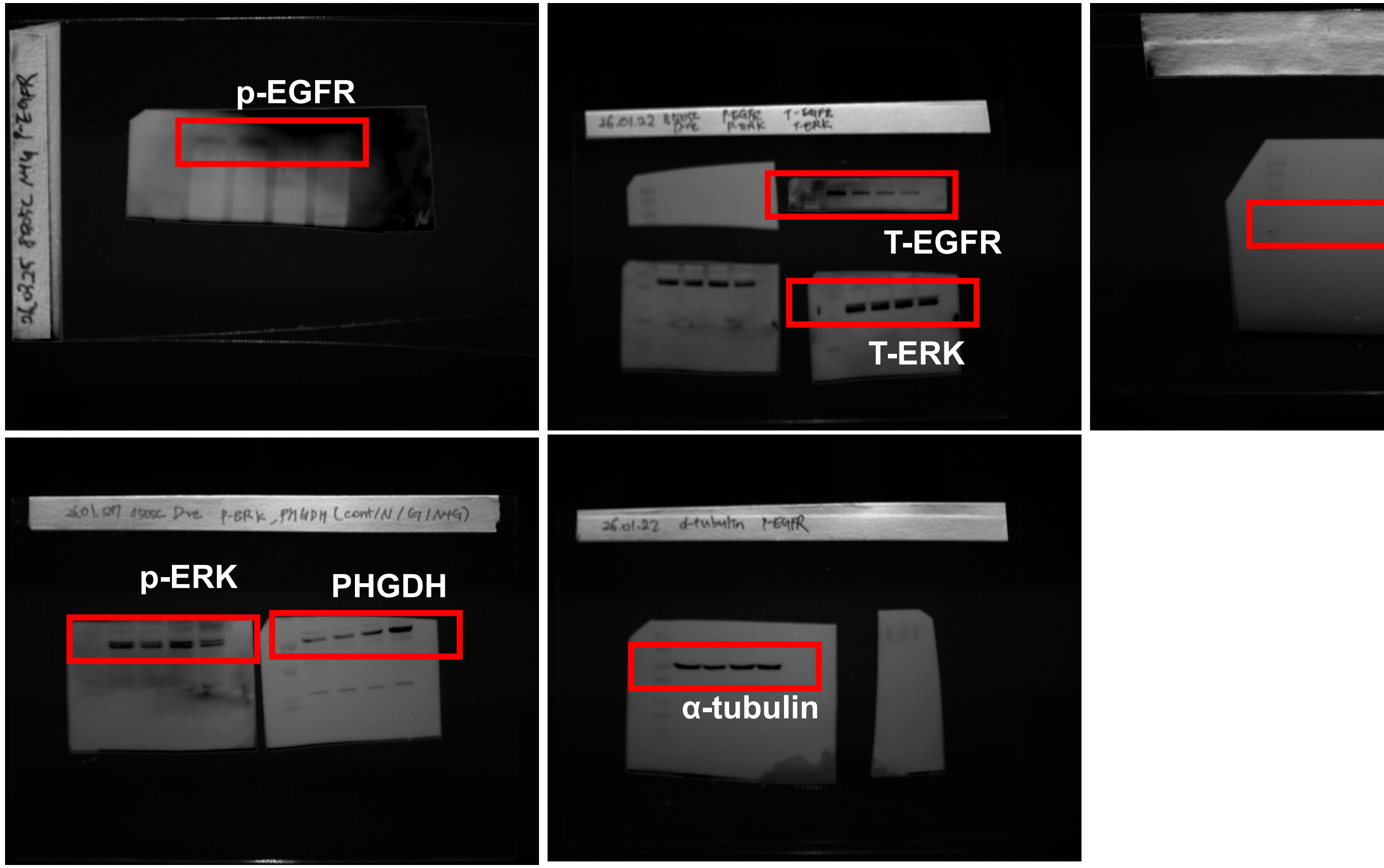

# Supple Figure 6

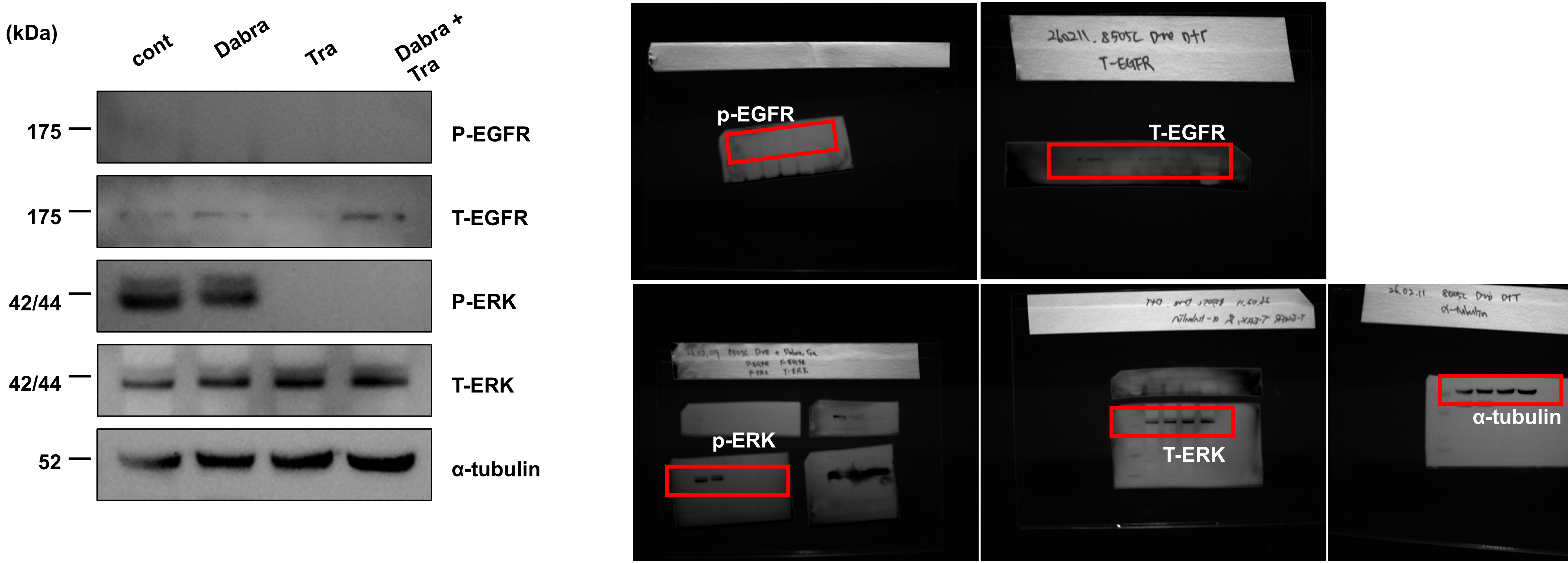

# Supple Figure 7

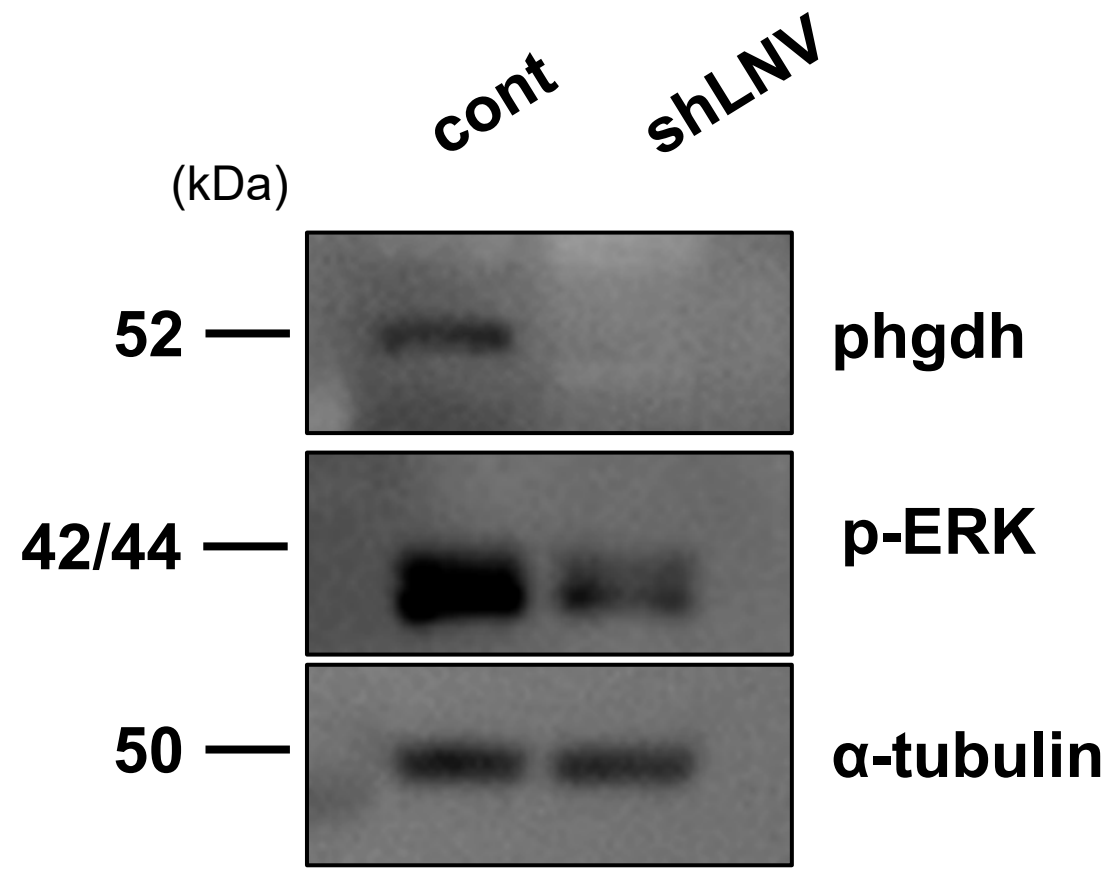


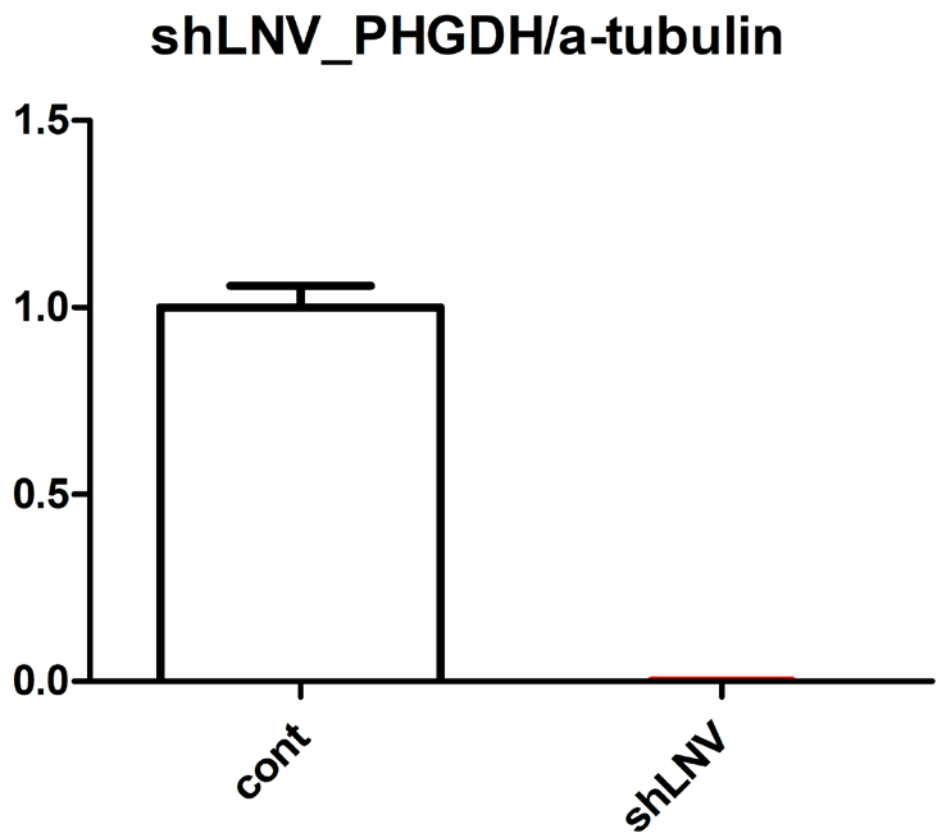


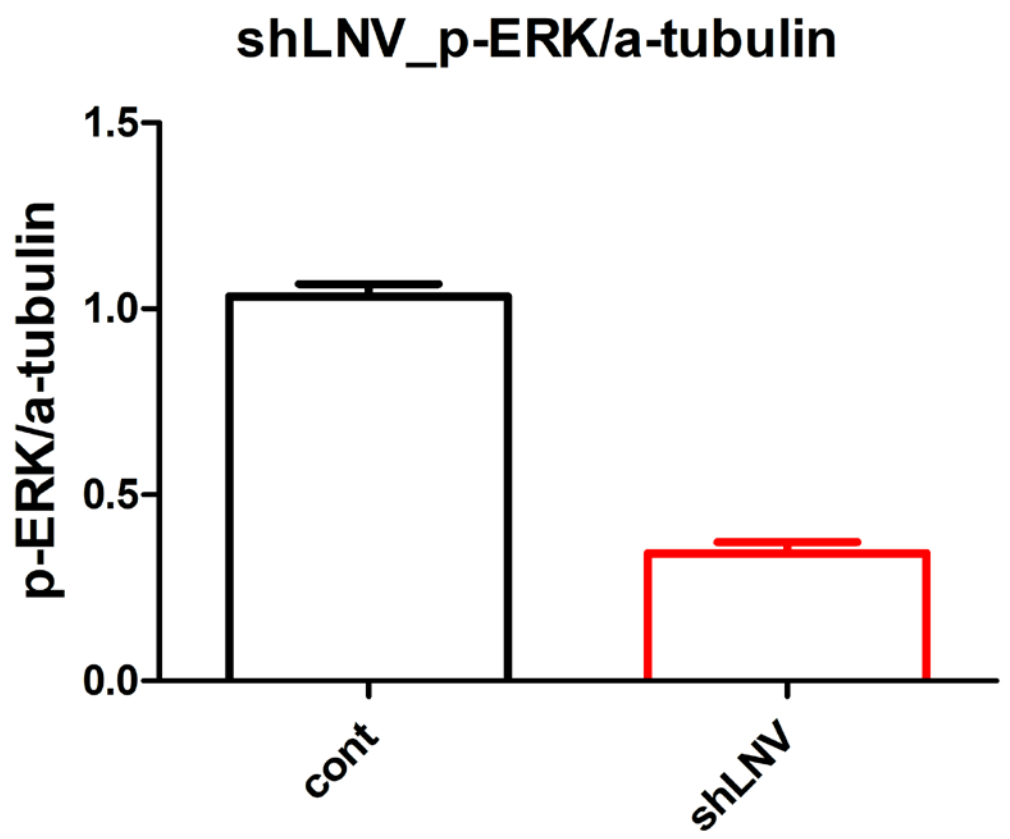


**1ST Dilution fold**

| | |
|---|---|
| **PHGDH** | **1:100** |
| **p-ERK** | **1:2000** |
| **t-ERK** | **1:2000** |
| **α-tubulin** | **1:5000** |

**PHGDH: Pico 사용**

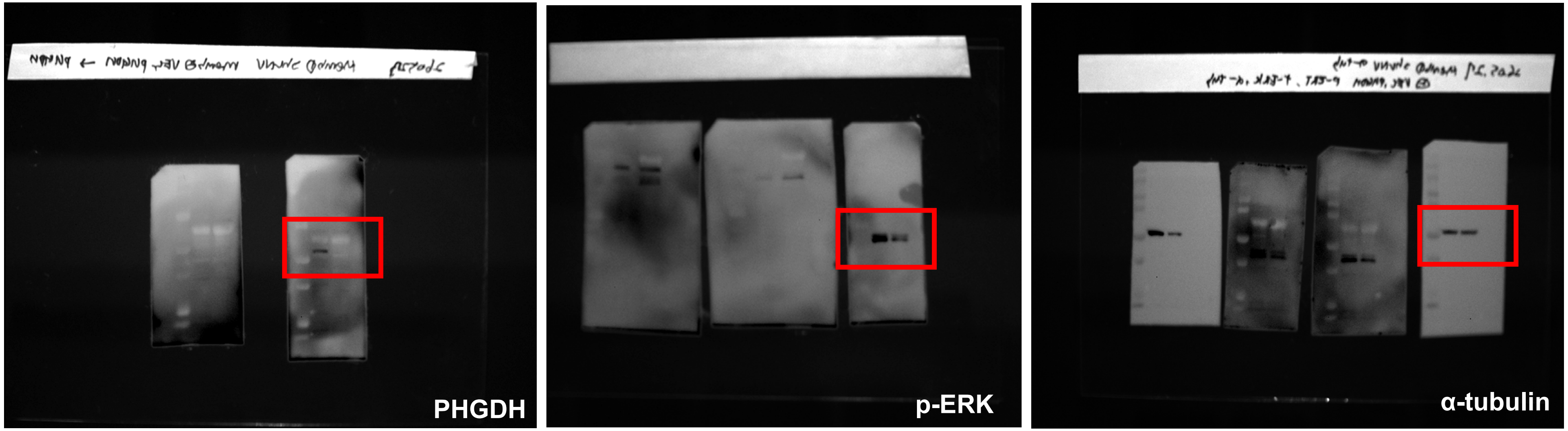

# Supple Figure 7

## western blot_

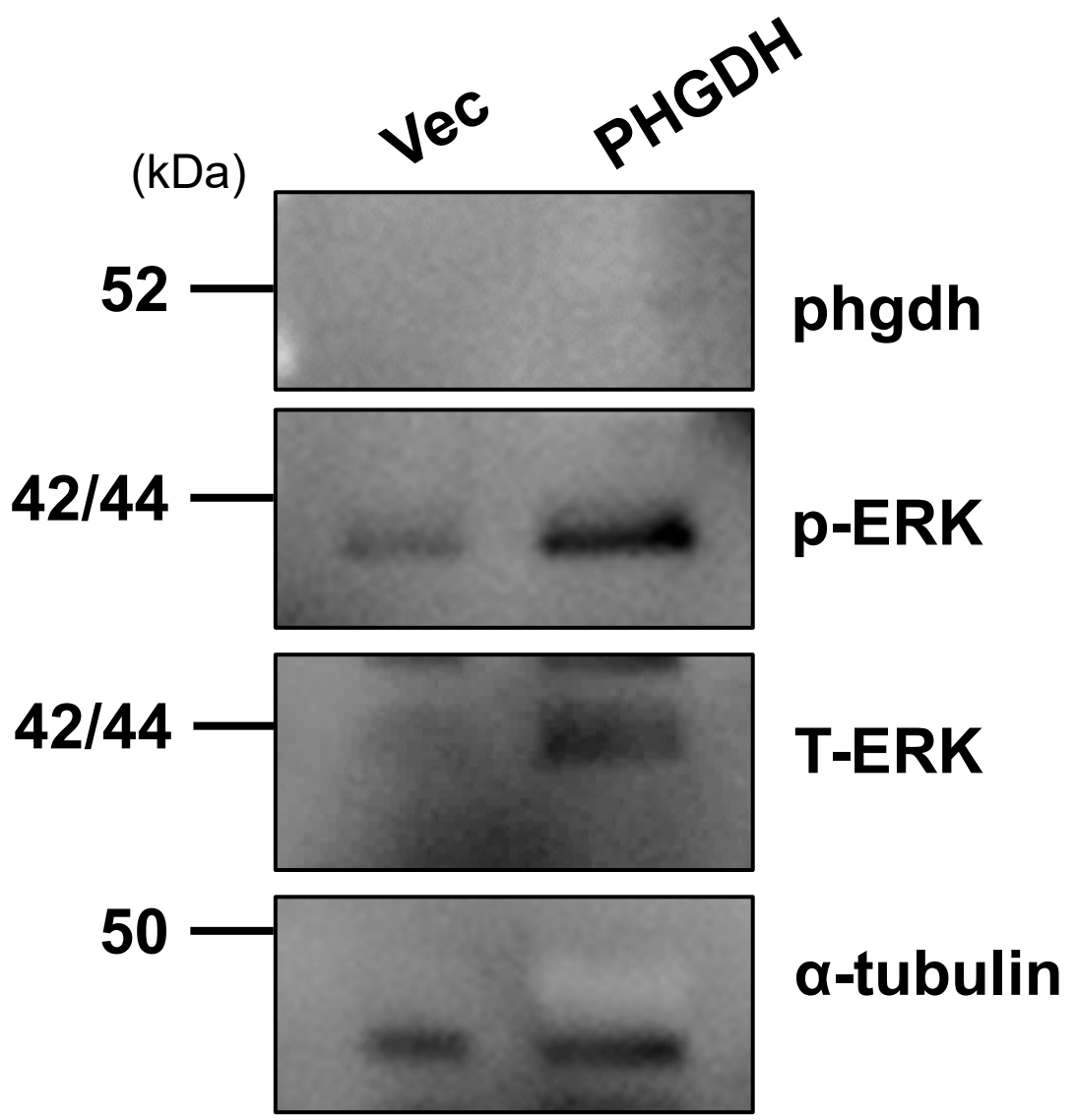


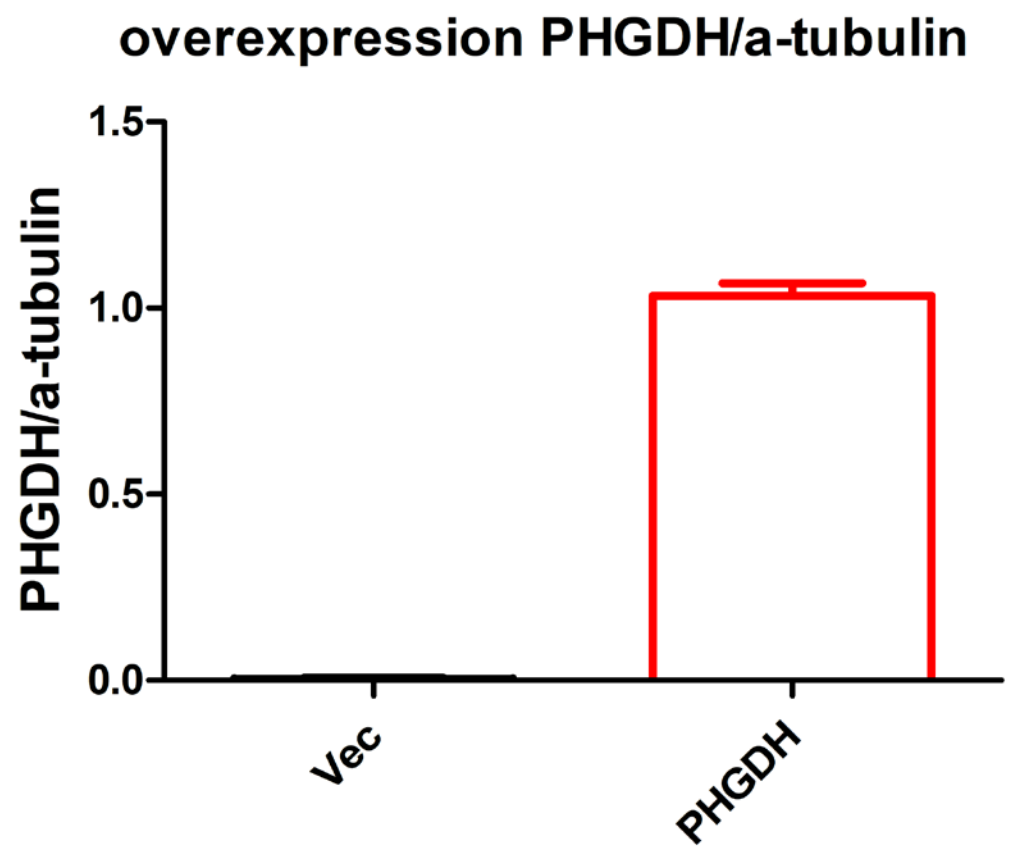


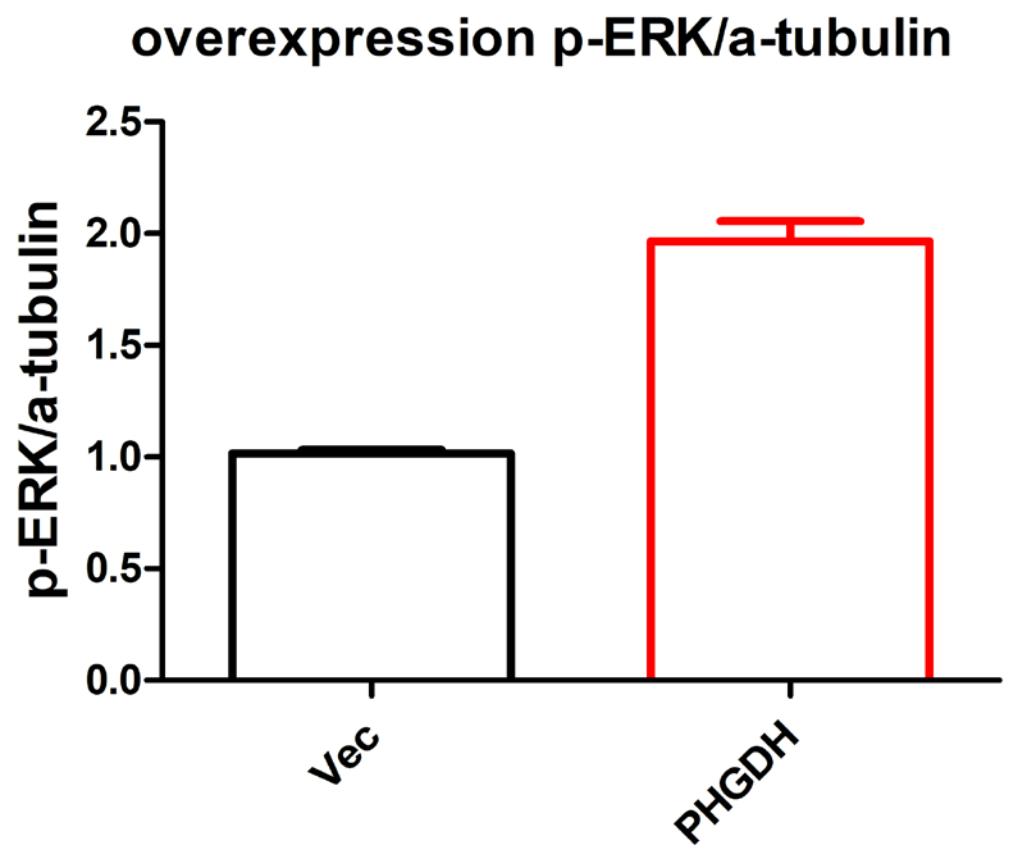


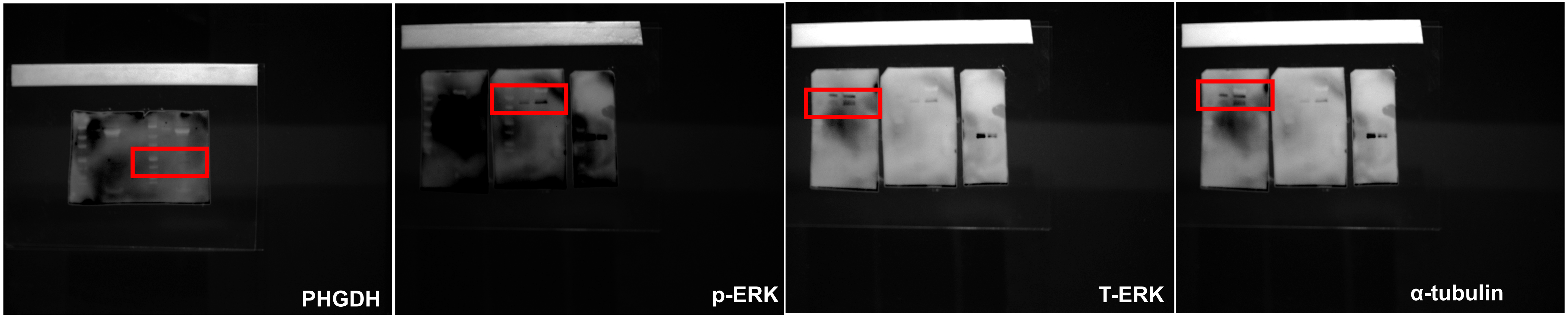

Supplement: Supplementary file 2 [file 41420_2026_3293_MOESM2_ESM.pdf]
