# Supplementary material for: PHGDH inhibition overcomes dabrafenib resistance through metabolic rewiring in BRAF V600E anaplastic thyroid carcinoma
Source: Cell Death Discov. 2026 Aug 15;12:452. doi: 10.1038/s41420-026-03293-7 (PMC13755417; doi:10.1038/s41420-026-03293-7)
Supplement: Supplementary file 3 [file 41420_2026_3293_MOESM3_ESM.pdf]

PHGDH inhibition overcomes dabrafenib resistance through metabolic rewiring in BRAF V600E anaplastic thyroid carcinoma

Sung Young Kim1†, Mi-Hyeon You2 †,*

†These authors contributed equally to this work and should be considered co-first authors.

Running title: PHGDH-mediated metabolic resistance in BRAF V600E ATC

Key words: anaplastic thyroid cancer, BRAF V600E mutation, Drug resistance, PHGDH, therapeutic vulnerability

Funding: This study was supported by the KonKuk University 2025.

Acknowledgement : We thank J-W Oh (Department of Dermatology, Severance Hospital, Cutaneous Biology Research Institute, Yonsei University College of Medicine, Seoul, 03722, Republic of Korea) for providing GEO data analysis presented in Fig. 4.

1: Department of Biochemistry, Konkuk University School of Medicine, Seoul 143-701, Republic of Korea, Tel.: 82-2-2049-6060; Fax: 82-2-2049-6192; E-mail: palelamp@kku.ac.kr

2: Department of Anatomy, College of Medicine, KonKuk University

268 Chungwon-daero Chungju-si Chungcheongbuk-do, 27478, Korea

Phone: +82-10-6602-6574, Fax:+82-43-840-3722, E-mail: shineth@kku.ac.kr

*Corresponding author and person to whom reprint requests should be addressed

ORCID

Mi-Hyeon You https://orcid.org/0000-0002-1318-2670

Supplementary Figure 1.

(A, B) Growth curves of dabrafenib-resistant cell lines derived from BCPAP cells in comparison with

the control BCPAP cells. In the absence of dabrafenib, the control cells grew statistically significantly (**p < 0.01) faster. In the presence of dabrafenib (1 μM), the resistant cells lines grew statistically significantly (**p < 0.01) faster. (C) Control and resistant 8505C cell lines were seeded (Initial seeding Number: 10,000 cells each), and colonies were stained at 7 days.

Supplementary Figure 2.

(A) Map of the RTK array.

(B) An increase in p-EKR in the dabrafenib-resistant cell line compared to the control. p-ERK quantification. The data are mean ± SD of three independent experiments (**p < 0.01).

Supplementary Figure 3.

To determine whether epithelial-to-mesenchymal transition (EMT) occurred in dabrafenib-resistant cell lines, N-cadherin, E-cadherin, and vimentin were detected by western blot analysis. The results showed no difference between resistant and control groups.

Supplementary Figure 4.

Expression patterns of stemness markers in dabrafenib-resistant ATC cells and response to PHGDH inhibition. (A) Heatmap illustrating the expression patterns of the extended stemness marker set. The heatmap displays hierarchical clustering of genes within the broad stemness marker set across control (C), dabrafenib-resistant (R), and PHGDH-inhibitor treated resistant (NCT-503) ATC cells. Genes are grouped into six distinct expression patterns (Groups 1-6) by consensus clustering. Expression values are z-scored across rows, with red indicating high expression and blue indicating low expression. (B) Differential expression of selected cancer stem cell-specific markers. Box plots show the expression levels of individual cancer stem cell markers, including ABCG2, BMI1, CD34, CD44, CTNNB1, EPAS1, EZH2, HIF1A, KDM5B, KLF4, MYC, NES, NOTCH1, BMP4, KRT14, PTCH1, TGFBR2, TNC, VEGFA, TET1, TBX3, and LRIG1 in control (C), dabrafenib-resistant (R), and PHGDH-inhibitor treated resistant (NCT-503) ATC cells. Statistical significance between groups is indicated by

individual p-values above the bars.

Supplementary Figure 5

Cell viability assay (MTT assay) showing the effect of NCT503 (15 μM) and dabrafenib (0.5 μM) alone or in combination on cell proliferation over 5 days. Cell viability was measured in 8505c. Data are mean ± SD (n = 3). The combination treatment (NCT503 + dabrafenib) significantly reduced cell proliferation at day 5 in comparison with the control group (**p < 0.01).

Supplementary Figure 6

(A–D) Effects of dabrafenib and trametinib on colony formation and MAPK signaling in dabrafenib-resistant ATC cells. Representative colony formation images following treatment with dabrafenib, trametinib, or combined dabrafenib plus trametinib are shown in (A). Quantification of colony numbers is presented in (B). Trametinib treatment markedly reduced colony formation compared with dabrafenib alone, while combined treatment further suppressed colony-forming capacity. Western blot analysis of EGFR/MAPK signaling pathways is shown in (C). The levels of p-EGFR and p-ERK were decreased following trametinib and combination treatment, whereas total ERK levels remained relatively unchanged. Quantification of the p-ERK/T-ERK ratio is presented in (D), confirming significant inhibition of MAPK signaling (* p<0.05, *** p<0.001).

(E–H) Effects of combined PHGDH and MEK inhibition on colony formation and ERK signaling. Representative colony formation assays following treatment with NCT503, trametinib, or combined NCT503 plus trametinib are shown in (E), with corresponding quantification in (F). Combined treatment further reduced colony formation compared with single-agent treatment. Western blot analysis demonstrated suppression of p-ERK signaling following NCT503 and trametinib treatment (G). Quantification of the p-ERK/T-ERK ratio is shown in (H), indicating effective inhibition of ERK activation by PHGDH and MEK blockade (* p<0.05; N.S., not statistically significant).

(I–J) Effects of combined PHGDH and EGFR inhibition on colony formation in dabrafenib-resistant ATC cells. Representative colony formation assays following gefitinib, NCT503, or combined NCT503

plus gefitinib treatment are shown in (I). Quantification of colony numbers is presented in (J). Combined PHGDH and EGFR inhibition markedly suppressed colony formation compared with gefitinib treatment alone, suggesting that PHGDH contributes to EGFR-mediated survival and proliferative signaling in resistant ATC cells (* $p<0.05$, ** $p<0.01$; N.S., not statistically significant).

The data are presented as the mean ± SD of three independent experiments (** $p<0.01$, and *** $p<0.001$).

Supplementary Figure 7

(A) Representative Western blot showing efficient knockdown of PHGDH in ATC cells transduced with shPHGDH compared with control cells.

(B) Quantification of PHGDH protein expression normalized to α-tubulin. Data are presented as relative expression levels compared with the control group.

(C) Rescue experiment in PHGDH-knockdown cells. Western blot analysis showing restoration of PHGDH expression following re-expression of PHGDH (shPHGDH+PHGDH) compared with vector-transfected shPHGDH cells (shPHGDH+Vec). Phosphorylated ERK (p-ERK), total ERK (T-ERK), and α-tubulin were analyzed.

(D) Quantification of PHGDH protein expression normalized to α-tubulin.

(E) Quantification of p-ERK levels normalized to total ERK. Re-expression of PHGDH significantly restored ERK phosphorylation in PHGDH-knockdown cells.

The data are presented as the mean ± SD of three independent experiments (** $p<0.01$, and *** $p<0.001$).
