# Supplementary material for: PHGDH inhibition overcomes dabrafenib resistance through metabolic rewiring in BRAF V600E anaplastic thyroid carcinoma
Source: Cell Death Discov. 2026 Aug 15;12:452. doi: 10.1038/s41420-026-03293-7 (PMC13755417; doi:10.1038/s41420-026-03293-7)
Supplement: Supplementary file 4 [file 41420_2026_3293_MOESM4_ESM.pdf]

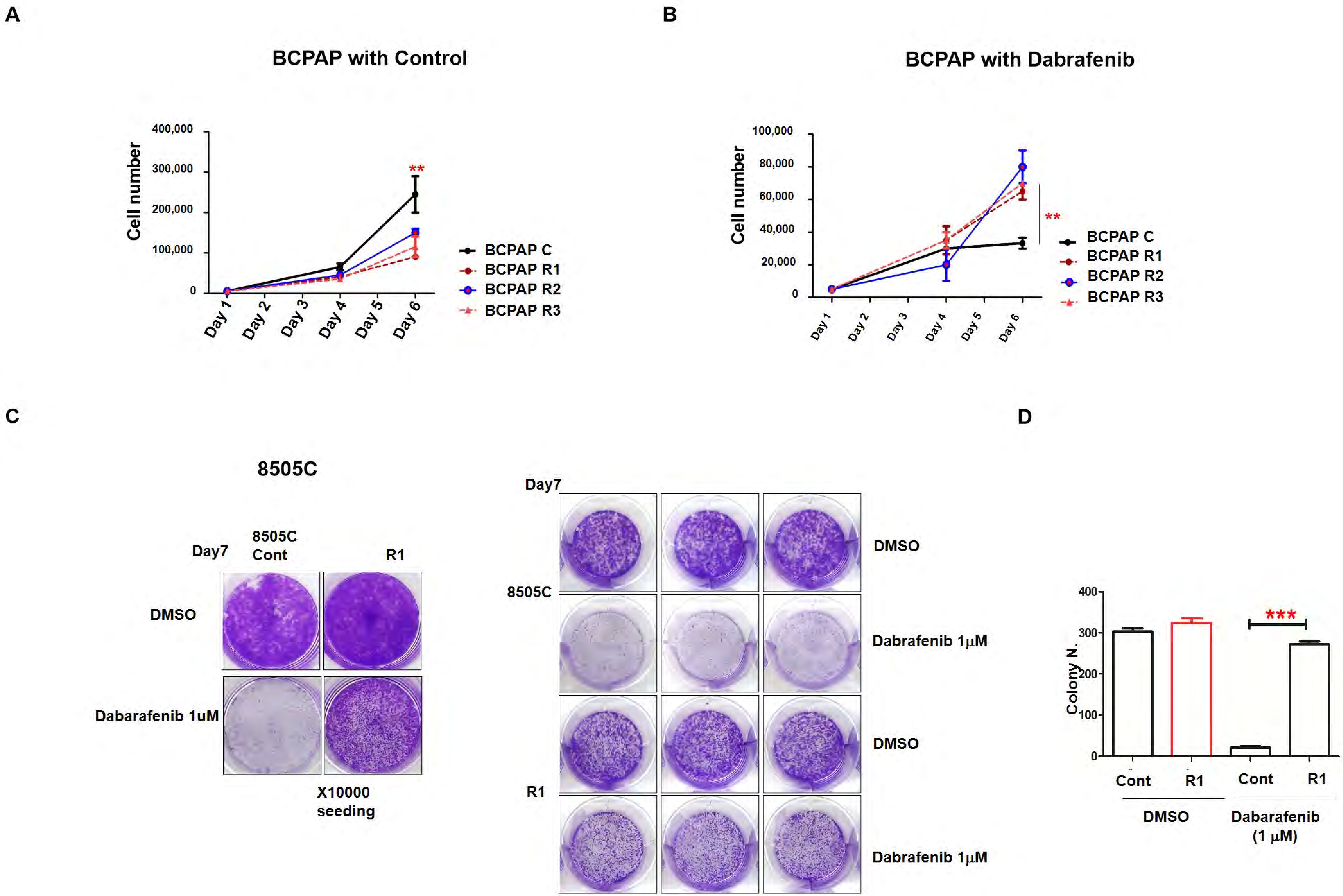
A
BCPAP with Control
Cell number
400,000
300,000
200,000
100,000
0
Day 1
Day 2
Day 3
Day 4
Day 5
Day 6
**
BCPAP C
BCPAP R1
BCPAP R2
BCPAP R3
B
BCPAP with Dabrafenib
Cell number
100,000
80,000
60,000
40,000
20,000
0
Day 1
Day 2
Day 3
Day 4
Day 5
Day 6
**
BCPAP C
BCPAP R1
BCPAP R2
BCPAP R3
C
8505C
Day7
8505C Cont
R1
DMSO
Dabarafenib 1uM
X10000 seeding
Day7
8505C
R1
DMSO
Dabrafenib 1μM
DMSO
Dabrafenib 1μM
D
Colony N.
400
300
200
100
0
***
Cont
R1
Cont
R1
DMSO
Dabarafenib (1 μM)
