# Supplementary material for: PHGDH inhibition overcomes dabrafenib resistance through metabolic rewiring in BRAF V600E anaplastic thyroid carcinoma
Source: Cell Death Discov. 2026 Aug 15;12:452. doi: 10.1038/s41420-026-03293-7 (PMC13755417; doi:10.1038/s41420-026-03293-7)
Supplement: Supplementary file 6 [file 41420_2026_3293_MOESM6_ESM.pdf]

A

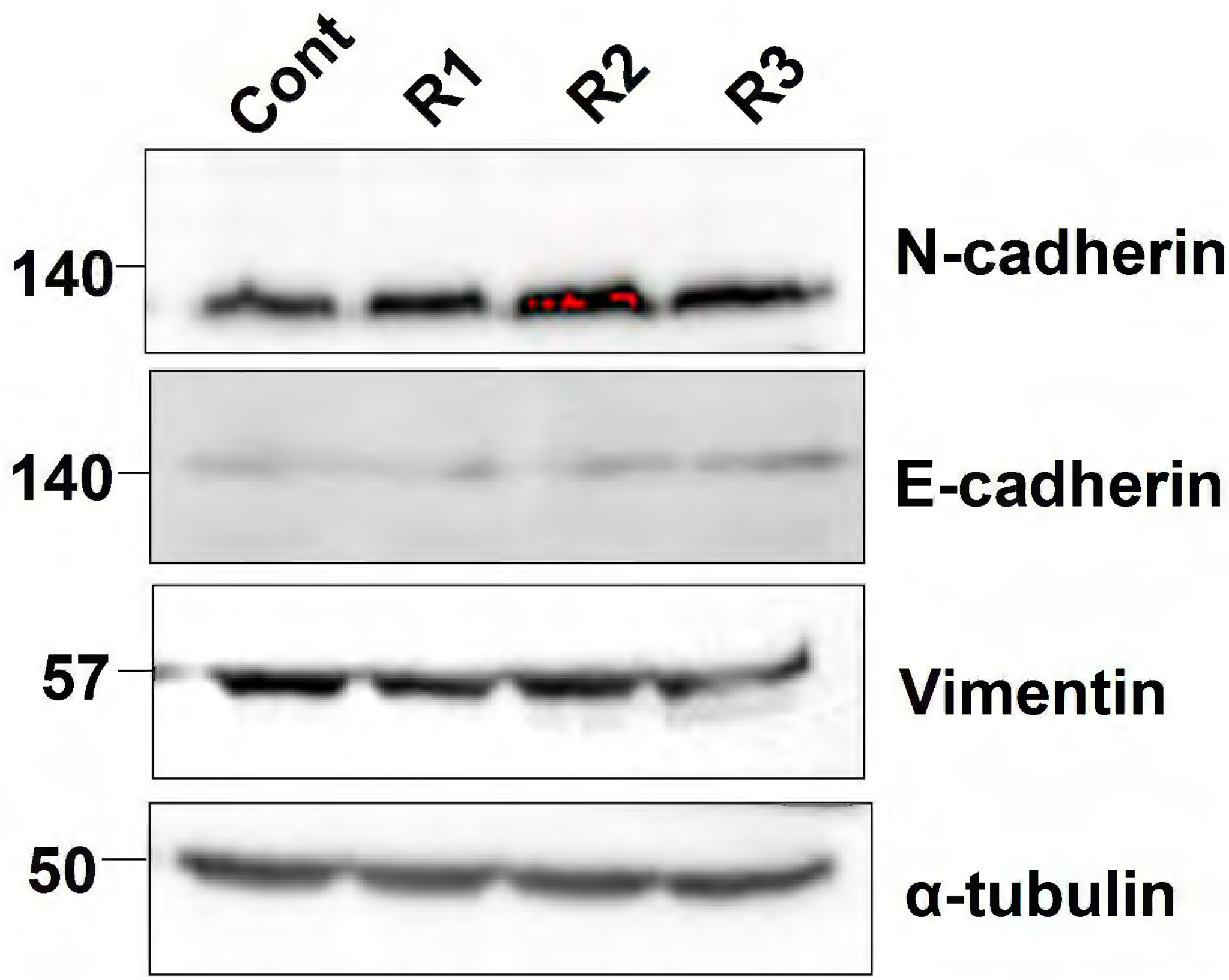
Cont
R1
R2
R3
140
N-cadherin
140
E-cadherin
57
Vimentin
50
α-tubulin
