# Supplementary material for: PHGDH inhibition overcomes dabrafenib resistance through metabolic rewiring in BRAF V600E anaplastic thyroid carcinoma
Source: Cell Death Discov. 2026 Aug 15;12:452. doi: 10.1038/s41420-026-03293-7 (PMC13755417; doi:10.1038/s41420-026-03293-7)
Supplement: Supplementary file 7 [file 41420_2026_3293_MOESM7_ESM.pdf]

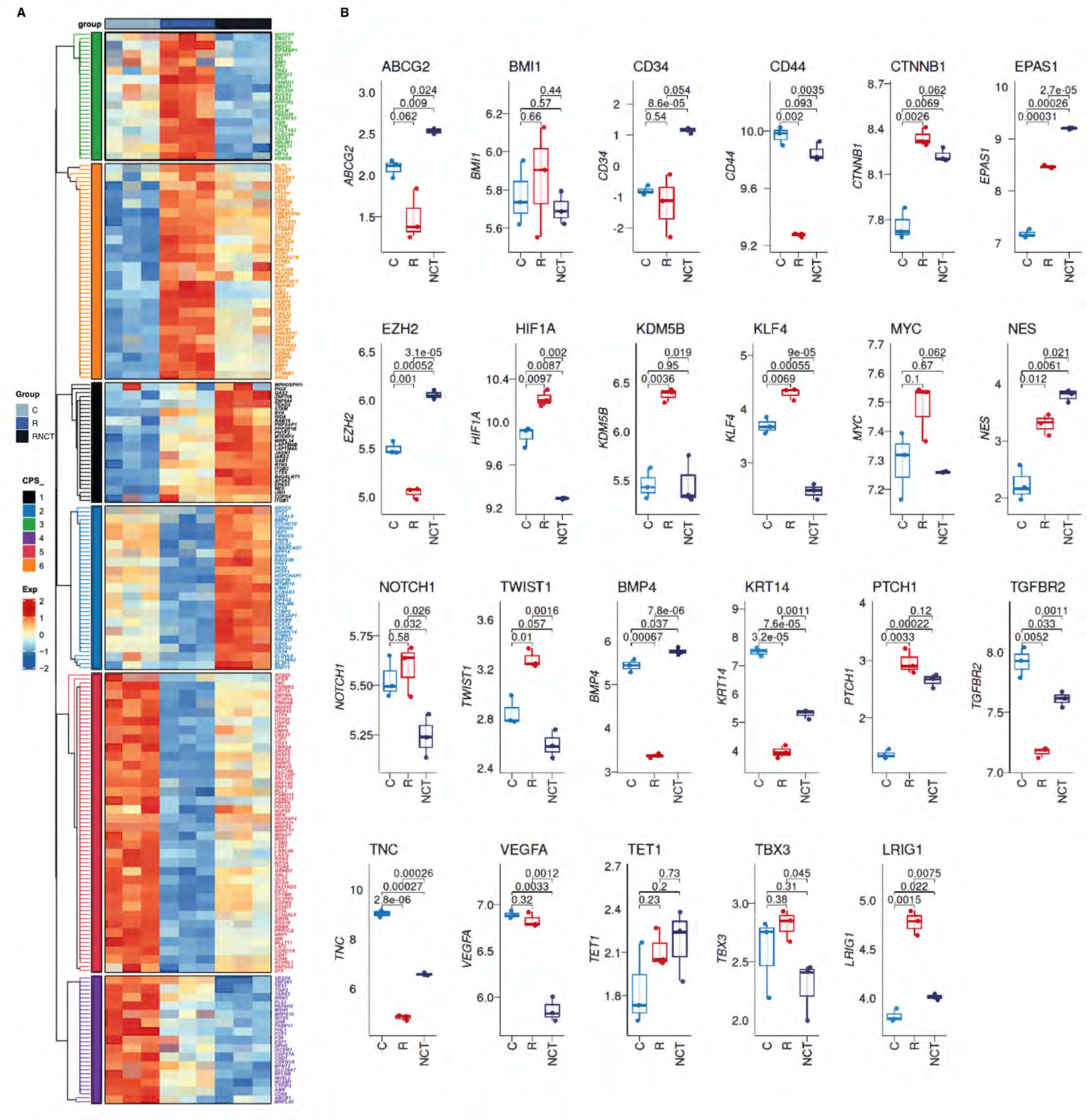
A
group
Group
C
R
RNCT
CPS_
1
2
3
4
5
6
Exp
2
1
0
-1
-2
B
ABCG2
0.024
0.009
0.062
BMI1
0.44
0.57
0.66
CD34
0.054
8.6e-05
0.54
CD44
0.0035
0.093
0.002
CTNNB1
0.062
0.0069
0.0026
EPAS1
2.7e-05
0.00026
0.00031
EZH2
3.1e-05
0.00052
0.001
HIF1A
0.002
0.0087
0.0097
KDM5B
0.019
0.95
0.0036
KLF4
9e-05
0.00055
0.0069
MYC
0.062
0.67
0.1
NES
0.021
0.0061
0.012
NOTCH1
0.026
0.032
0.58
TWIST1
0.0016
0.057
0.01
BMP4
7.8e-06
0.037
0.00067
KRT14
0.0011
7.6e-05
3.2e-05
PTCH1
0.12
0.00022
0.0033
TGFBR2
0.0011
0.033
0.0052
TNC
0.00026
0.00027
2.8e-06
VEGFA
0.0012
0.0033
0.32
TET1
0.73
0.2
0.23
TBX3
0.045
0.31
0.38
LRIG1
0.0075
0.022
0.0015
C
R
NCT
