# Supplementary material for: PHGDH inhibition overcomes dabrafenib resistance through metabolic rewiring in BRAF V600E anaplastic thyroid carcinoma
Source: Cell Death Discov. 2026 Aug 15;12:452. doi: 10.1038/s41420-026-03293-7 (PMC13755417; doi:10.1038/s41420-026-03293-7)
Supplement: Supplementary file 8 [file 41420_2026_3293_MOESM8_ESM.pdf]

A

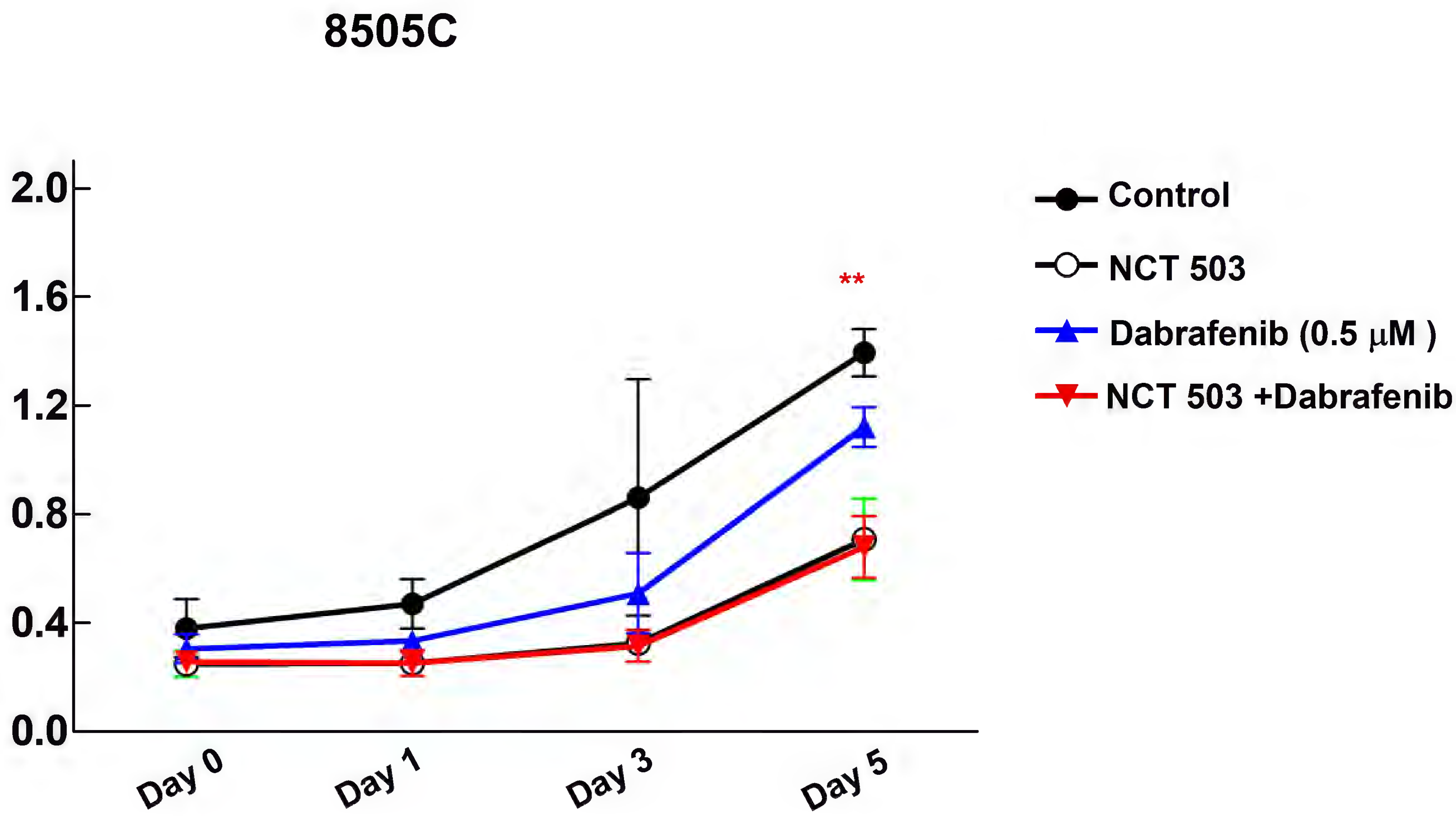

8505C
2.0
1.6
1.2
0.8
0.4
0.0
Day 0
Day 1
Day 3
Day 5
**
Control
NCT 503
Dabrafenib (0.5 μM)
NCT 503 +Dabrafenib
