# Supplementary material for: PHGDH inhibition overcomes dabrafenib resistance through metabolic rewiring in BRAF V600E anaplastic thyroid carcinoma
Source: Cell Death Discov. 2026 Aug 15;12:452. doi: 10.1038/s41420-026-03293-7 (PMC13755417; doi:10.1038/s41420-026-03293-7)
Supplement: Supplementary file 9 [file 41420_2026_3293_MOESM9_ESM.pdf]

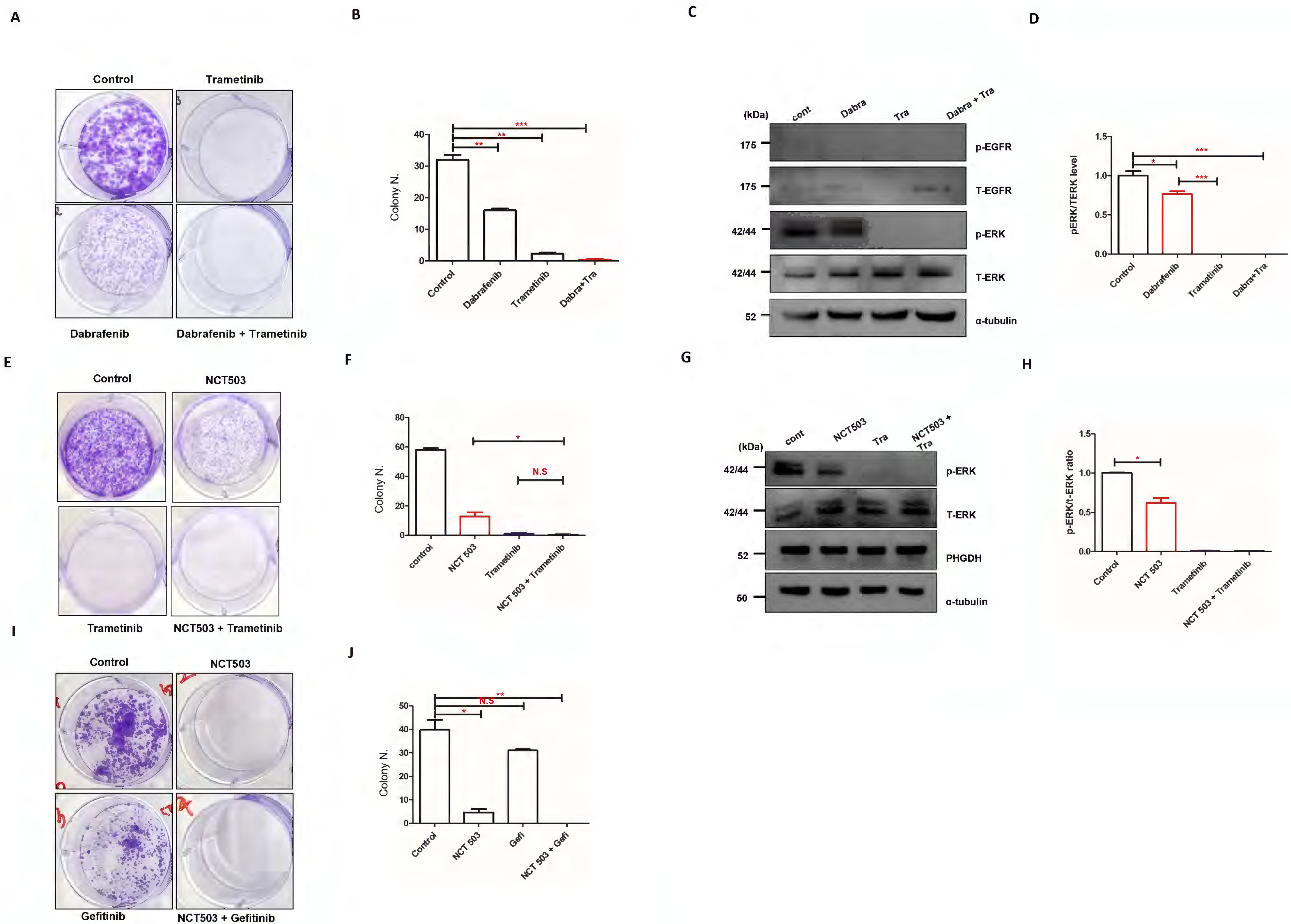
A
Control
Trametinib
Dabrafenib
Dabrafenib + Trametinib
B
Colony N.
Control
Dabrafenib
Trametinib
Dabra+Tra
C
(kDa)
cont
Dabra
Tra
Dabra + Tra
175
175
42/44
42/44
52
p-EGFR
T-EGFR
p-ERK
T-ERK
α-tubulin
D
pERK/TERK level
Control
Dabrafenib
Trametinib
Dabra+Tra
E
Control
NCT503
Trametinib
NCT503 + Trametinib
F
Colony N.
N.S
control
NCT 503
Trametinib
NCT 503 + Trametinib
G
(kDa)
cont
NCT503
Tra
NCT503 + Tra
42/44
42/44
52
50
p-ERK
T-ERK
PHGDH
α-tubulin
H
p-ERK/t-ERK ratio
Control
NCT 503
Trametinib
NCT 503 + Trametinib
I
Control
NCT503
Gefitinib
NCT503 + Gefitinib
J
Colony N.
N.S
Control
NCT 503
Gefi
NCT 503 + Gefi
